# Assessing sustained high-heat multi-hazard events through earth observation and impact chain analysis in southeast UK

**DOI:** 10.1016/j.isci.2026.115423

**Published:** 2026-03-24

**Authors:** Erin Mills, Kay Smith, Annie Winson, Luke Bateson, Roxana Ciurean

**Affiliations:** 1 British Geological Survey, Lyell Centre, Research Avenue South, Edinburgh, UK; 2 British Geological Survey, Nicker Hill, Keyworth, Nottinghamshire, UK

**Keywords:** earth sciences, environmental science, environmental monitoring

## Abstract

Sustained high-heat events are increasing in the UK, intensifying risks to health, infrastructure, ecosystems, and water resources. This study investigates whether earth observation (EO) data can identify environmental preconditioning factors and candidate thresholds that signal emerging multi-hazard conditions. Using the 2022 southeast UK heatwave, we analyze climate variables and EO-derived indicators—including land-surface temperature, soil moisture, vegetation stress, and ground motion—at regional and event-specific scales, supported by a hazard impact catalog. Impact-chain analysis reveals how heat, drought, vegetation stress, wildfires, flash flooding, and subsidence interact as compounding and cascading hazards. We find that soil-moisture deficits, elevated surface temperatures, and ground-motion anomalies frequently precede hazardous events, indicating their potential as early-warning signals. This multi-source synthesis provides a semi-automatable workflow and EO-based thresholds benchmarked against a multi-year baseline, offering broader value for monitoring and forecasting high-heat multi-hazard risks under accelerating climate change.

## Highlights

• Sustained high heat in 2022 drove widespread multi-hazard impacts across southeast UK
• We found evidence that EO anomalies aligned with emerging hazard conditions
• Evidence from EO analyses can underpin future tools for emerging multi-hazard risk

## Graphical abstract

**Figure:**
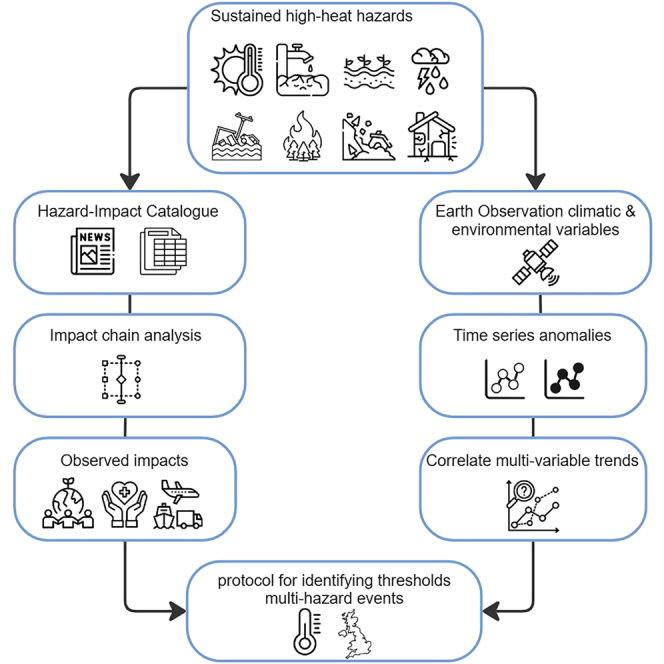

## Introduction

Climate change is having a large impact on our global societies presenting a fundamental threat to physical and ecological environments and therefore impacting the human environment.^1^ One of these threats is heatwaves, whereby the natural environment is subject to prolonged periods of abnormally hot, dry weather. The environmental impact of heatwaves results in the exacerbation of prolonged dry conditions resulting in the development or a worsening of drought, reduction in water availability, ecological damage through increased vegetation stress or reduced water quality, and an increased wildfire risk.^2^ Heatwaves can impact populations affected, stressing health due to reductions in air or water quality, as well as through secondary impacts associated with an increase in demand for power or through losses in agriculture or marine life sectors.^3^ The continuation of heatwave conditions can lead to a cascade of interacting geological and hydro-meteorological-hazards such as flash flooding, subsidence, and landslides, which have a widespread impact on the human, infrastructure and ecology.

**Figure 1:**
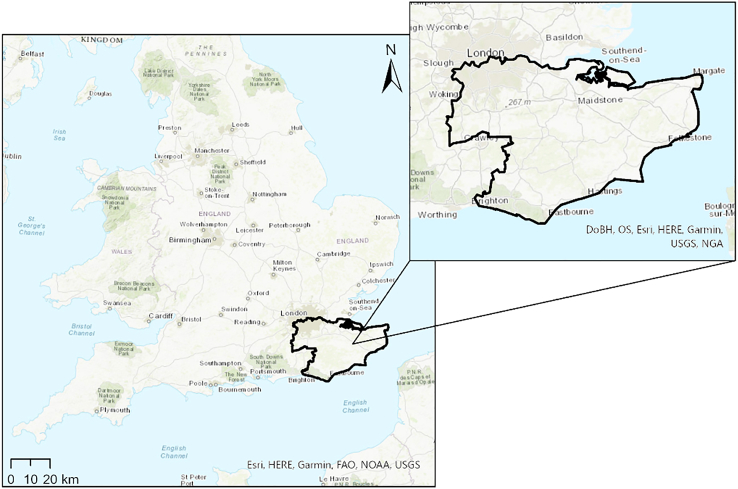
Map highlighting the southeast UK, with the study area focused on the counties of Kent, East Sussex and south Greater London Boundary is displayed in ArcGIS Pro on topographic basemap image. (Source: DoBH, OS, Esri, HERE, Garmin, USGS, NGA, FAO, and NOAA).

Over the past 10 years, unprecedented hot and dry conditions have resulted in hazards and multi-hazard interactions that have not previously been recorded in the UK. There is a pressing need for a better understanding of these increasingly anomalous climatic conditions and their subsequent impacts on the human and natural environments. This case study focuses on the multi-hazards that have affected the Southeast region of the UK, an area including the counties of Kent and East Sussex as well as part of the Greater London Authority (Figure 1). This region has particularly been the locus for hazards associated with sustained high temperatures, with extreme events recorded in 2018, 2022,^4^ and most recently in 2025. As such, the southeast UK is a prime research laboratory in which to study the interrelated effects of climate change and sustained high-heat events.

Hazards experienced in this region have included heatwaves, drought, shrink-swell driven subsidence, wildfires, rockfalls, and urban heat island. Paradoxically, some of these drying hazards result in a higher probability of flooding and an increased number of precipitation-triggered landslides in this region later in the year, due to induration of the clay-rich soils that have been dried out or subjected to wildfires. When rain does fall on these soils, the decreased infiltration rate means that water cannot be absorbed into the soils and therefore it flows across the surface, generating flash flooding and in some cases debris flows. The increasing occurrence of these multi-hazards in areas where they have previously been less common can be attributed to climate change.^5^ Future projections of the effect of climate change predict the long-term increase in the frequency of hotter, drier summers and wetter winters in Northern Europe^6^^,^^7^^,^^8^ leading to a higher incidence of the types of hazards we have identified in this study, and exposing a larger number of people and assets to these multi-hazard interactions.

Southeast UK is the most densely populated region in the UK, home to over 19 million people despite covering roughly 8% of the country’s total land area.^9^ This region features a diverse range of land uses including agricultural and national designated landscapes (i.e., Kent Downs), as well as dense urbanization (including transportation, telecommunications, and energy infrastructure) due to its proximity to London, the UK capital. There is also a target to build more than 200,000 new homes in the region by 2040^10^ alongside an expected population growth of over 400,000 between 2018 and 2028.^11^ This increase will place pressure on key resources such as water, whose use is already reaching capacity. Over the period covered in this study (2022), the UK Environment Agency (EA) advised water restrictions across England due to low reservoir levels and increased demand. By the end of August 2022, 17 of the 18 English water companies had activated drought plans, five of which introducing temporary bans on water use.^12^ Some of these restrictions were extended and still in effect until September 2023^13^ due to persistent below average precipitation.

In this study, we investigate the potential use of EO-derived data for monitoring and understanding the implications of sustained high-heat events in the UK. The investigation focuses on the complex and interacting nature of multiple hazards related to sustained high-heat, namely heatwaves, drought, wildfires, thunderstorms, flash flooding, landslides, and subsidence occurring in 2022. To achieve this, we (1) review information from both official and gray literature (in this study, gray literature, refers to information produced on all levels of government, academic, business, and industry in electronic and print formats not controlled by commercial publishing. These could include local and regional news reports, impact related documents from public bodies, policy briefings and/or departmental reports^14^) sources to build an evidence-based catalog of events and empirically driven impact chains; (2) develop time-series of satellite-based earth observation (EO) data and their derived parameters over the last two decades to identify trends and tipping points in environmental and climatic variables that indicate the potential onset of a multi-hazard cascade; and (3) investigate the potential of *in situ* data to complement existing satellite-based EO data and their derived parameters.

The core questions addressed in this study are as follows:

1. How do spatially and temporally compounding events, such as droughts and heatwaves, lead to or else amplify the occurrence and impact of other types of hazards, particularly in regions not previously associated with such type of hazards?
2. Can satellite-based EO data and their derived parameters help identify trends and tipping points leading to cascading or amplifying effects in a multi-hazard event, which could lead to the development of tools and methods for monitoring and early warning?
3. Is it possible to identify candidate thresholds in observable environmental changes that indicate the onset of compounding or cascading multi-hazard events?

The availability of satellite-based EO data from the early 1980s to the present has created the opportunity to understand the Earth system through the derivation of parametric products and their application to earth/environmental science. This long baseline of data and its derived parameters provides an ability to understand the condition of the environment before multi-hazard events occur and monitor the changes after, prior to the onset of accelerated climate change. As the temporal range of available satellite data and derived parameters ever increases, their application to the understanding of multi-hazard events is also extended. It is hoped that this pragmatic, testable workflow will increase opportunities for future forecasting of the impacts of climate change on multi-hazards occurrence, while also increasing opportunities to explore the statistical robustness of derived relationships and in turn feed into opportunities to develop early warning solutions based on our understanding of EO data.

To reduce future losses, it is essential to understand how multi-hazard events (cascading, amplifying, and compounding) from sustained high temperatures increase the exposure of population and infrastructure, especially those that are already vulnerable or at risk due to pre-existent socio-economic disparities, aging infrastructure, or limited access to resources. Compound events involve the combination of multiple natural hazards, such as droughts, heatwaves, or heavy rainfall, leading to complex interactions and amplified impacts. In contrast, cascading events refer to a sequence of events triggered by an initial event, often with significant impacts, where subsequent events are directly or indirectly related to the trigger event. Cascading events create a cause-and-effect chain reaction, while compound events involve concurrent or sequential hazard occurrences that can amplify their overall impact.^15^ Both types of events pose challenges for disaster management due to their complexity and potential for significant consequences (https://eo4multihazards.gmv.com/faq/). These interactions are challenging to predict and can result in more significant and destructive consequences than individual hazards alone. For example, the co-occurrence of droughts and heatwaves can amplify their impact. Advancing this knowledge will enable more effective collaboration with stakeholders to aid in improving the monitoring and forecasting efforts of future multi-hazard events, supporting enhanced decision-making and risk management.

### UK sustained high-heat event of 2022

In the UK, the Met Office often uses the term “high-heat” as a descriptive precursor for the declaration of a heatwave. Depending on the location in the UK, the threshold for a heatwave to be declared varies between 25°C and 28°C where the daily maximum air temperature exceeds this value for three consecutive days.^16^ In this study we define a “high-heat” event as a period of unusually elevated temperatures for the time of year that may not be sustained long enough or reach the intensity required to meet the official heatwave criteria.

During the high-heat events that occurred in the summer of 2022, 5 heatwave episodes were officially declared in the southeast region of the UK. Indeed, 2022 was the warmest recorded in the 364-year Central England Temperature (CET) series, which began in 1659, with an annual mean daily temperature of 13.9°C. This was 1.1°C warmer than the 1991–2020 average, with elevated conditions experienced throughout the year.^17^ Overall, the summer months of June to August were ranked 4th warmest on record.^18^ In addition, 2022 was the fifth driest summer season since the 1890s.^12^ The persistent high-heat and below average summer precipitation posed a significant risk to public safety and resulted in 2,985 excess deaths.^19^ In response to this, the Met Office worked closely with the United Kingdom Health Security Agency (UKHSA) to ensure the timely issuance of health alerts. These heat health alerts (HHAs) have been operational since 2004, having been created for the heatwave plan for England in response to the 2003 European heatwave.

**Figure 2:**
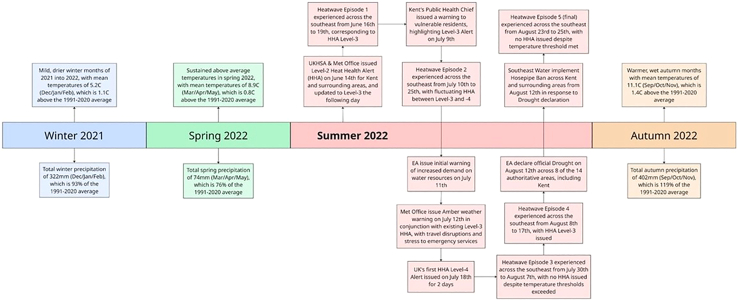
Timeline of the unfolding of summer 2022 heatwave episodes across UK

A crucial component of this study is the “heat episodes” of 2022, which refer to periods of excess mortality relating to continuous high-heat, as defined by the Met Office and UKHSA. They are defined as any day on which level 3 HHA was issued in at least one region in England, with a day on either side of the alert included to allow for lagged effects on temperature mortality. HHA level 3 is the second highest alert level, which indicates impacts are likely to be felt across the whole health service, with potential for the whole population to be at risk; non-health sectors may also start to observe impacts.^20^ Where episodes overlapped, they have been counted as a single episode. These episodes are described in Figure 2 below. The hottest day on record during 2022 accounted for 638 more deaths than normal, followed by the next day which claimed an additional 496 lives.

Many parts of the southeast began to experience high air temperatures in mid-June, with parts of London reaching 28°C for three consecutive days, over the threshold set by the Met Office defining a heatwave. This prompted the issuance of a level 3 HHA for London and southeast England more broadly by the UKHSA. The high-heat generated thunderstorms and air temperatures dropped overnight by 10°C–15°C on 17^th^ June into 18^th^ June.^21^ In response to these events, the Met Office issued yellow warnings for thunder and lightning. At this time there were also reports in both official and gray literature sources of several landslides and rockfalls linked to the warm dry weather across the UK such as Seaford Head (Sussex) and Sidmouth Cliffe (Devon), with warnings issued to the public.^22^ The relationship between rockfalls and high temperatures may be explained by the expansion of rock due to heat with subsequent cooling causing pre-existing cracks in the rocks to widen and additional cracks to form. As the temperature fluctuates this increases the risk of the rocks failing and causing rockfalls and topples.^23^^,^^24^

Temperatures began to rise again in early to mid-July, prompting the Met Office to issue an extreme heat warning for the 17^th^ July, which was then extended into the following week. Increased rockfalls were reported with resultant warnings in response to the sustained high-heat and lack of precipitation.^25^ Water resources came under stress as reservoirs, such as Ardingly (West Sussex), Bough Beech (Kent), and Hanningfield (Essex), began reporting below normal water levels due to a combination of increased usage/demand and lack of precipitation both prior to and during this second heatwave episode.^26^ During this heatwave, wildfires were observed at Dartford, Kent,^27^ and Wennington, east London,^28^ as vegetated areas were tinder dry. These occurred near populations where homes and adjacent areas had to be evacuated and infrastructure networks near these wildfires were disrupted. Wildfires increased in occurrence from 17^th^ July onwards (a total of 50 documented until the end of September), particularly in response to the sustained high surface temperatures, drying ground conditions and summer winds enabling them to spread quickly. This caused damage to domestic gardens and public green spaces, to areas of wildlife and conservation and significant damage to crops, such as on Lenham Heath, Kent,^29^ resulting in lower crop yield.

This sustained high-heat event in July was the first time that temperatures exceeded 40°C in the UK since records began, the new record was measured at 6 locations: 40.3°C in Coningsby and Waddington in Lincolnshire, 40.2°C at Heathrow, and areas in London measuring 40.2°C in St. James Park, 40.1°C in Kew Gardens and 40.0°C in Northolt.^30^ An additional 46 Met Office weather stations recorded temperatures of over 38.7°C, surpassing the previous UK record. The sustained high-heat was experienced across the entire UK, not just in the southeast where this study is focused. On 18^th^ July, UKHSA issued their first level 4 HHA for the whole of England, which indicates significant risk to life to the population and indicated severe impacts would be expected across all sectors.^31^ The temperatures in England exceeded 38°C for two consecutive days, while the highest temperatures were recorded in Lincolnshire on the 19^th^ July, reaching 40.3°C and exceeding the previously recorded maximum temperature by 1.6°C.^32^

Rising temperatures and limited precipitation resulted in drought conditions as early as mid-July, which continued into August. By this time, the EA had announced drought in eight of their 14 areas (districts) across England.^33^ This was later extended to additional areas in late August, as conditions worsened and due to the ongoing lack of precipitation. As a result of the drought, several water companies implemented limited water use by imposing hose pipe bans. Despite these measures, an increasing number of reservoirs across the UK experienced decreases in water levels. Lindley Wood and Thruscross reservoirs in North Yorkshire reported below average levels on 13^th^ July and Colliford reservoir in Cornwall dropped by 15% overall in 2022.^34^ Colliford reservoir is the water source for 225,000 households and so this level of water reduction had the potential to severely impact a large population. There were also reports of smaller water bodies running dry from 19^th^ July, e.g., Aysgarth Falls on the River Ure in North Yorkshire.^35^

Due to these persistent conditions, vegetation became very dry and stressed resulting in the spread of wildfires, despite fire and rescue services dampening vegetation in an attempt to reduce the presence of hotspots. At least 24,316 wildfires were recorded across fire services in England from June to August, representing almost four times the 6,213 wildfires over the same three-month period in 2021.^36^ First responders such as fire and rescue services responded to 620,758 incidents in the UK in the year ending September 2022, a 16% increase compared to the previous year. Of these incidents, there were 185,437 fires, a 28% increase compared with the previous year. This increase in fire incidents can be attributed to the 44% increase in secondary fires and the 63% increase in primary outdoor fires following the hot, dry summer. Indeed, there were 21,241 outdoor fires reported in July 2022 (compared to 7,251 in 2021) and 22,513 outdoor fires in August 2022, an increase of more than 3-fold when compared to 7,156 in the previous August.^37^ Secondary fires are small, typically outdoor fires that do not result in casualties or property damage, such as grass or bin fires, and require only a minor response. Outdoor fires is a broader category that includes both these secondary fires and more serious primary outdoor fires that result in significant damage, casualties, or require a larger emergency response. With increasing risk, comes increased strain on water resources, operational demand, and sustained investment.^38^

During this time there were several Met Office warnings for thunderstorms, which occurred as temperatures dropped across the country. Flash flooding was forecast for Kent due to heavy precipitation on previously inundated surfaces and the potential for ignition of fires in dry vegetation caused by lightning was also highlighted. Parts of the country experienced flash flooding due to the intense precipitation associated with the thunderstorms, most notably southwestern areas including Devon and Cornwall. There were also episodes of flash flooding in London, High Beech (Essex) and Frittenden (Kent) between 17^th^ and 18^th^ July. Surface water flooding caused damage to homes and businesses, severe travel disruptions, and increased pressure on local first responders who had already been responding to wildfires.^39^ Landslides and rockfalls were also reported throughout this period across the UK, both in response to the drying ground and heavy onset of precipitation associated with the thunderstorms after rapid cooling of temperatures. Anecdotal evidence suggests that subsidence increased during this sustained heat event resulting in approximately 18,000 insurance claims, with insurers during this time reporting claims of £219 million because of ground displacement, with the average claim approximately £9,600.^40^ This is a rise of 299% in claims between 2013 and 2022.

## Results

### Impact chain analysis

**Figure 3:**
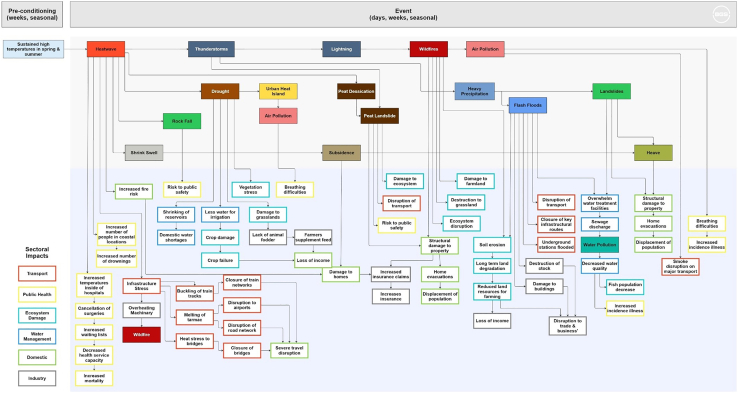
Impact chain representing the hazard cascades and associated impacts in southeast UK during the summer heatwave episodes of 2022

A general analysis across southeast UK indicates that the main impacts of sustained high temperatures lead primarily from heatwave and droughts cascading to other secondary hazards, affecting sectors such as public health and transport (Figure 3). As these hazards progress through time, the number of impact categories increases, meaning more areas of society are affected and response and mitigation efforts become increasingly complex across sectors. This observation coincides with other research outputs relating to the event.^41^^,^^42^^,^^43^

An example following on from the heatwave-drought interaction is vegetation stress followed by wildfires. Vegetation stress occurs throughout the system due to a lack of water availability. Dependent on the cascade, this can have a multitude of impacts across sectors, such as crop damage and failure, habitat damage, or can lead to secondary hazards such as soil erosion and wildfires.^44^ Wildfires result in considerable stress on fire and rescue services, in addition to disruption across transport networks (i.e., roads and rail).

**Figure 4:**
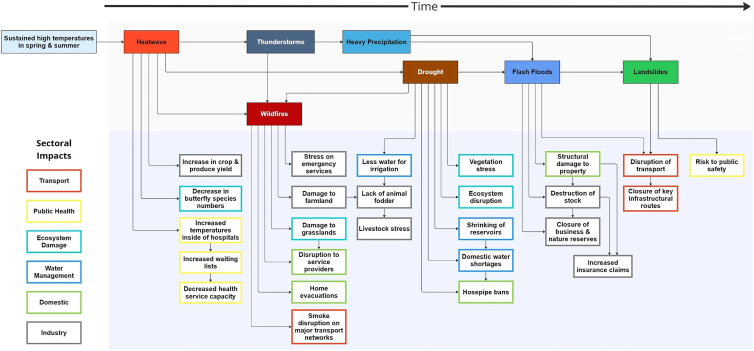
Impact chain representing the hazard cascades and associated impacts specific to the county of Kent during the summer heatwave episodes of 2022

When focus is placed on the hazard interaction across the county of Kent (Figure 4), over 50 individual events were geospatially identified and assigned to a specific thread of the impact chain. This section of the analysis focused on the impact pathway linking heatwaves and droughts to subsequent interactions with wildfire and/or flash flood.

The heatwave caused increased temperatures to be experienced inside hospitals in southeast UK, above predefined safe limits. This meant that procedures were canceled and resulted in longer waiting lists for patients and in turn a reduced health service capacity,^45^ further impacted by the critical infrastructure such as large data centers that came under increased stress due to the additional cooling requirements from the increased temperatures.^46^

This trend was seen across other parts of the country and not just specific to the county of Kent. Initially there were positive implications of sustained high-heat, particularly for farmers who were able to obtain crop surplus, which resulted in a decreased price for fresh produce (e.g., strawberries and cherries) in southeast UK. This positive impact was quickly overturned as drought brought damage to crops and farmlands,^47^ and heat stress brought to livestock.^48^ Drought was prominent across the county of Kent, with the lack of precipitation resulting in many local reservoirs reporting below average levels for that time of year.^49^ In late August a long-term hosepipe ban was implemented to preserve water resources, but the lack of water resulted in associated agricultural stress due to restricted regular irrigation during the persistent heat episodes. In conjunction with the drought, wildfires caused additional damage to crop and farmlands. This damage to farmlands was reported in the context of preventative measures taken by local farmers, creating fire breaks by cutting crops near active wildfires to prevent further spread and active area dampening.

Later in the year, after a drier than average spring and summer, the prolonged dry conditions resulted in a prevalence of harder, impervious soils. That meant when precipitation increased considerably, potentially due to thunderstorm activity, there was overpressure on the drainage system from surface runoff. This flash flooding resulted in transport and travel disruption on both road and rail networks, with damage to businesses and properties and an increased stress on emergency services.

### Pattern recognition from EO data for the county of Kent

**Figure 5:**
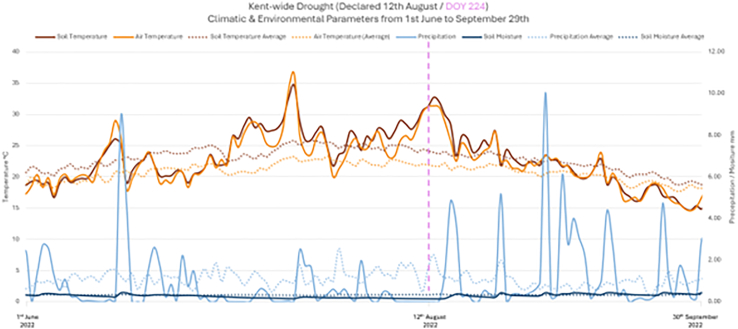
Comparative time-series for climatic and environmental parameters extracted from ECMWF data for the county of Kent Solid curves indicates 2022 data, compared with dotted curves indicating the average for 2000–2023 (excluding 2003 and 2018). Declared drought is also indicated (dashed vertical line). Contains modified Copernicus Climate Change Service Information (2022) ECMWF data.^50^

The representative time-series of the Kent-wide climate variables of air temperature, soil temperature, precipitation and soil moisture from 2022 were compared to the average climate condition for the 2000–2021 period (Figure 5) for a 100-day window from 1^st^ June to 29^th^ September.

During the sustained high temperature event of 2022, the county of Kent experienced drought which was declared by the EA on 12^th^ August (marked by the vertical dashed line in Figure 5). A general overview of the detail observed in the climate data around this time suggested that the air temperature was significantly higher than the average between July and August 2022, where July showed the greatest departure from the norm. Both air and soil temperatures remained above average between 4^th^ July (39 days prior to official drought declared) until 7^th^ September (26 days after official drought declared). Precipitation remained low throughout June and July and below average between 2^nd^ July and 1^st^ September (41 days prior to and up to 20 days after official drought declared), except for the occurrence of four large precipitation events after drought declaration before the temperature declined below average. Soil moisture was below average between 2^nd^ July and 25^th^ August (41 days prior to and up to 13 days after official drought declared). The drought across the county of Kent can therefore be broadly characterized by 65 days of air temperature above the average, 61 days of below average precipitation and 54 days of below average soil moisture. Closer examination of the time-series data revealed emerging patterns in the climate characteristics.

Both the air and soil temperatures were raised from 9^th^ August (3 days prior to official drought declared), consistently above average, but an upwards trend was seen from 26^th^ July (17 days prior to official drought declared). Air temperature peaked between 12^th^ and 14^th^ August (on the day and for 3 days after official drought declared) and soil temperature peaked on 13^th^ August (1 day after official drought declared). Both temperatures fell sharply on 7^th^ August (5 days after official drought declared) and were coupled with a noticeable precipitation event experienced on 16^th^ August (4 days after official drought declared). More specifically, the climate data highlighted rapid decline in air and soil temperature that corresponded with a peak in above average precipitation repeatedly across the time-period (1^st^ June to 29^th^ September). After 14^th^ September, air and soil temperatures remained below average until the end of the time-period.

Below average precipitation was observed from 26^th^ June through to 31^st^ August (47 days prior to and up to 19 days after official drought declared) with exception to several above average events that corresponded with reported thunderstorms that brought heavy precipitation and a sudden rapid drop in air temperatures. Little to no precipitation was experienced between 2^nd^ and 19^th^ July (41–24 days prior to official drought declared), and on the 23^rd^ July and 14^th^ August (20 days prior to and up to 2 days after official drought declared). The sharp increase from 14^th^ to 16^th^ August (2–4 days after official drought declared) was associated with a thunderstorm that led to flash flooding in Frittenden (flash flood event #1047). There was another observable spike in precipitation between 24^th^ and 25^th^ August (12–13 days after official drought declared), with an associated sharp decrease in air and soil temperature.

Soil moisture was observed as lower than average between 3^rd^ July and 16^th^ August (40 days prior to and up to 4 days after official drought declared) and increased to match the average by a precipitation event on 16^th^ August. The soil moisture then dropped between 20^th^ and 24^th^ August, which can be associated with peaks in both air and soil temperatures on the 18^th^, 22^nd^, and 24^th^ August.

We also analyzed the spatial patterns in climate variables in relation to the landscape characteristics of Kent using the satellite-derived ground motion time-series from the European Ground Motion Service (EGMS), BGS Geology 50K Bedrock and Superficial geology layers, and the aggregated UKCEH 2022 land cover map.

**Figure 6:**
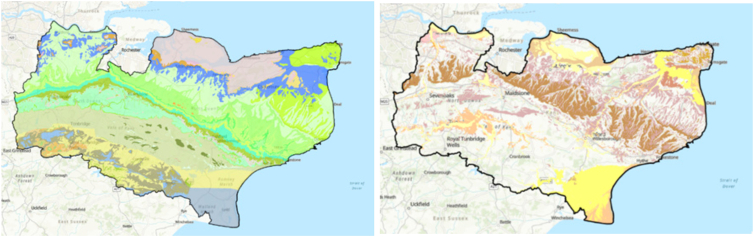
Geology of the county of Kent from BGS Geology 50K (left) Bedrock Geology and (right) Superficial geology (geological unit legend available via the GeoIndex Onshore Viewer) Data are displayed on ArcGIS Pro world basemap topographic layer (Source: ESRI, TomTom, Garmin, FAO, NOAA, USGS, © OpenStreetMap contributors, and the GIS User Community).

Ground motion hazards can be linked to sustained periods of high-heat. This is especially true for areas where the underlying geological deposits (either bedrock or superficial) are susceptible to volume change as they dry out. In shrink-swell clays, clay minerals shrink during periods of high sustained heat. This volume change can damage property and infrastructure.^51^^,^^52^ The bedrock and superficial geologies for the county of Kent are shown in Figure 6. The bedrock geology consists predominantly of chalk (24.1%), mudstone (14.3%), and sandstone and siltstone (23%). Clay is present in a small area of the county. The superficial deposits in Kent consist predominantly of clay, silt, sand, and gravel, of which 71.2% contain clay. For full unit detail, please refer to the BGS GeoIndex Onshore Viewer (https://mapapps2.bgs.ac.uk/geoindex/home.html). The aggregated UKCEH land cover for Kent is dominated by vegetated surfaces (82.6%).

**Figure 7:**
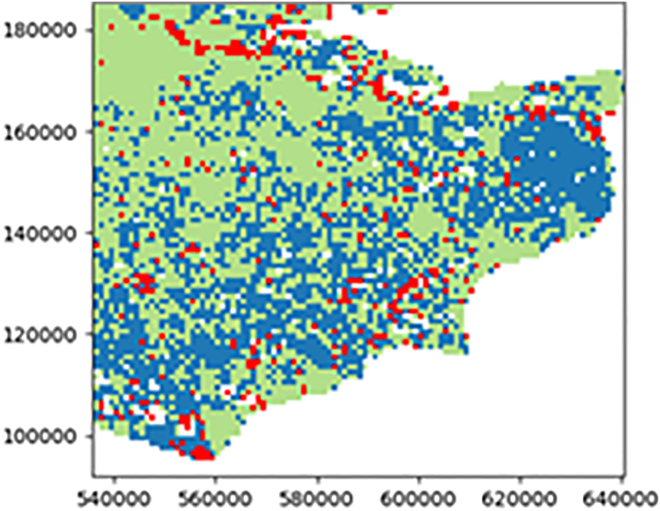
Dominant ground motion pattern from EGMS InSAR 2018–2023 time-series data for southeast UK, produced using a BGS internal tool The ground motion for each 1 km × 1 km grid cell is categorized into subsidence (red), stable (green), uplift (pink), and non-linear (blue). Generated using European Union’s Copernicus Land Monitoring Service Calibrated 2019–2023 (vector), Europe, yearly data. DOI: https://doi.org/10.2909/8889e0a7-a6df-47a8-b4e7-a9cb32cbbf6b.

Assessment of the satellite-derived ground motion during the high-heat event was performed by investigating the average velocities over the time-period and examining the time-series patterns specifically for areas of clay that were susceptible to shrink-swell. This was achieved using an internal BGS tool that examined the time-series ground motion of every InSAR point within a spatial subset, at 1 km × 1 km grid cell in this study (BGS, in preparation). The tool identified stable and unstable points based on a velocity threshold (±1.889 mm/yr) and, for the latter, classified the motion according to the pattern over time in relation to break-points, a break-point being where a change in motion occurred such as from subsidence to uplift. The patterns were categorized into linear stable, linear subsidence, linear uplift and non-linear, potentially with a background seasonal component. The dominant ground motion pattern for southeast UK, focusing on the county of Kent, is shown in Figure 7.

**Figure 8:**
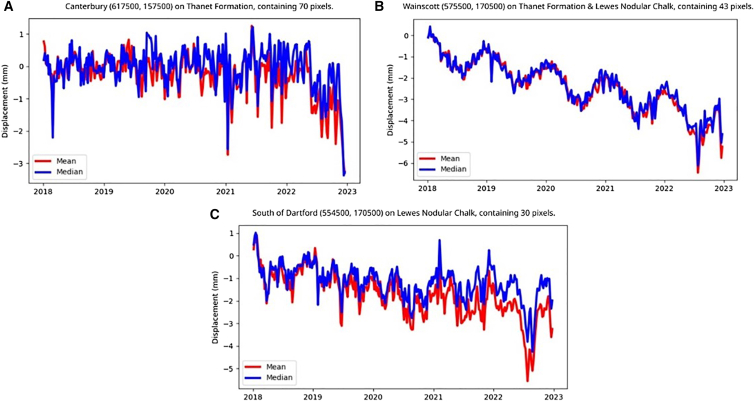
Ground displacement time-series plots in the county of Kent for predominant bedrock geologies A 1 km × 1 km grid cell of (A) sand, silt, and clay, (B) chalk, sand, silt, and clay and (C) chalk. Generated using European Union’s Copernicus Land Monitoring Service Calibrated 2019–2023 (vector), Europe, yearly data. DOI: https://doi.org/10.2909/8889e0a7-a6df-47a8-b4e7-a9cb32cbbf6b.

Changes in ground motion relating to the 2022 period of sustained high-heat were investigated for a selection of InSAR points in southeast UK (Figure 8). Spatial and temporal correlations were examined from areas where deposits of clay are present in both the superficial (clay with flints and slope derived head deposits) and bedrock (London clay). In Kent, the overall ground motion trends for urban area A near Canterbury was one of stability with a noticeable subsidence of ∼ −2.5 mm in the latter part of 2022. The geology of area A consisted of a bedrock of sand, silt, and clay overlain by clay and silt superficial deposits. While the overall trends for urban area B near Wainscott and area C near Dartford were one of subsidence, the trends showed additional subsidence of ∼ –2 mm in mid-2022 for area B and ∼ –3 mm in mid-2022 for area C. The geology of area B consisted of a bedrock of chalk, sand, silt, and clay and superficial deposits of clay, silt, sand, and peat, while area C consisted of a bedrock of chalk with a small portion of superficial deposits of clay, silt, and sand gravel.

The timing of the observed ground motion suggested a link to the hotter, drier conditions experienced in southeast UK. Additional factors that can modify this response include surface loading and bedrock or superficial geology properties; effects related to clay and chalk or low groundwater levels are already indirectly captured through the sustained high-heat and associated drying.

#### Pattern recognition analysis of EO-derived parameters

**Figure 9:**
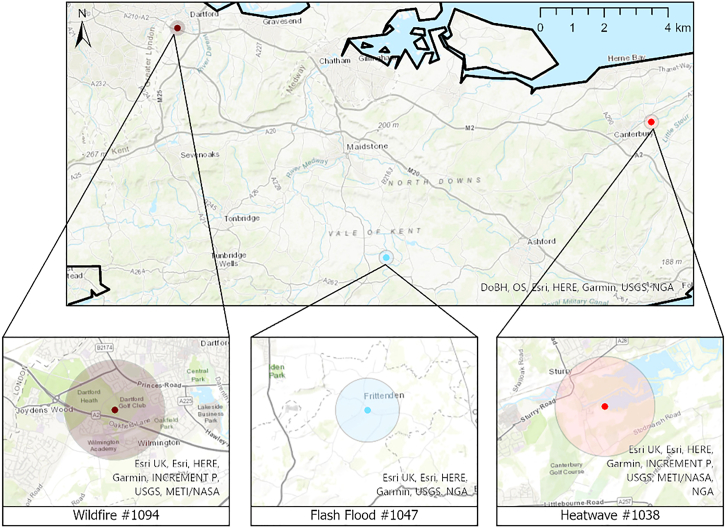
Location of specific 2022 events from the HIC: heatwave #1038, wildfire #1094 and flash flood #1047 Data visualized in ArcGIS Pro using the basemap world imagery layer (Source: Esri, Maxar, Earthstar Geographics, CNES/Airbus DS, and the GIS User Community).

Geospatial time-series statistics extracted from the daily and monthly aggregate climate data variables and environmental parameters were analyzed to identify patterns associated with the 2022 hazard events during the high sustained temperature period across the county of Kent. This section focuses on specific hazards representing the heatwave, wildfire, and flash flood events (Figure 9) occurring between 1^st^ June and 30^th^ September 2022.

**Figure 10:**
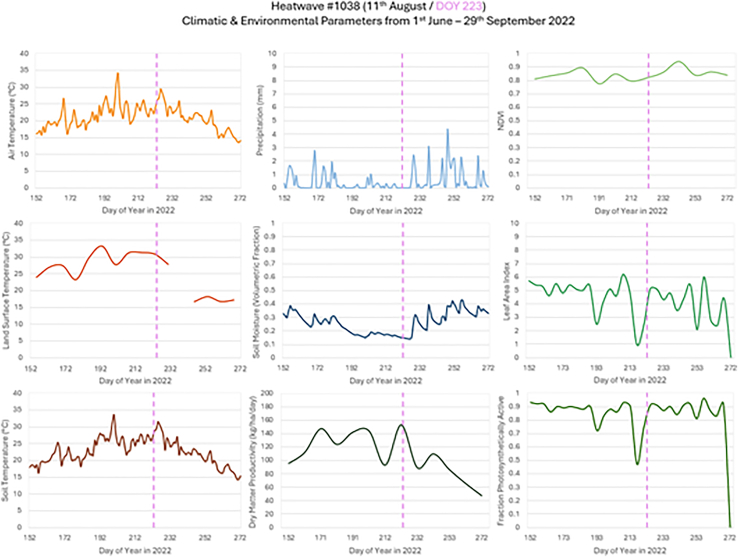
Time-series of climatic and environmental parameters for the period 1^st^ June–29^th^ September 2022 for a heatwave event in Kent (HIC event #1038) Local heatwave announcement indicated with dashed vertical line. Contains data from modified Copernicus Climate Change Service Information (2022) ECMWF^50^ and Copernicus Land Monitoring Service products.

Each specific event was analyzed to identify patterns, potential trigger points and candidate thresholds in the climate variables and environmental parameters, in conjunction with the corresponding segment of the impact chain. These analyses are discussed in relation to the bedrock and superficial geologies, aggregated land cover, and ground motion. Results from pattern recognition are presented for selected events from the HIC, including the heatwave (Figure 10), wildfire (Figure 12), and flash flood (Figure 14) that occurred in Kent in 2022.

#### Pattern recognition for a specific heatwave event

The prolonged high-heat and dry conditions attributed to a heatwave near Canterbury (HIC event #1038) resulted in an algal bloom, which impacted the water quality where over 1,000 fish died in a nearby fishery lake (31 acres, but occupying just 10% of the event buffer) due to lack of oxygen. The EA loaned six aerators to pump oxygen back into the water to save the remaining fish population. Climate variables and environmental parameters associated with this heatwave were examined (Figure 10).

Air temperatures were consistently above the 17°C average between 30^th^ June and 17^th^ September, with soil temperature consistently above 18°C during the same period. The air and soil temperatures did not meet the Met Office heatwave threshold of 28°C before the heatwave was officially declared. However, land surface temperature values were consistently above 28°C during the 48 days prior to the declaration and 5°C warmer than both air and soil temperatures during this time and followed a similar trend in warming pattern. The lack of cloud-free land surface temperature data were evident by the gap in late August, which coincided with the cooling period of both air and soil temperatures. This gap made it difficult to identify meaningful trends in land surface temperature after the heatwave event announced on 11^th^ August. Through these 4 months, daily precipitation remained below 2 mm, rising above this on a few occasions associated with periods of rainfall. The lack of precipitation leading up to the heatwave event correlated with a gradual decrease in soil moisture and a short lag in soil moisture increase related to the periods of rain. Due to the lack of precipitation, vegetation became stressed which was evident through the decrease in both leaf area index (LAI) and fraction of photosynthetically active radiation (FPAR). While normalized difference vegetation index (NDVI) values were expected to fall during the period due to the high temperature and lack of precipitation causing vegetation stress, values remained high (>0.8) in this location and were higher than for other similar events in the area. As the NDVI peak occurred between 31^st^ August and 10^th^ September, this may be associated with the reported algal bloom. The dry matter productivity (DMP) showed a pronounced fluctuation before the event, potentially in response to particular phases of vegetation growth rate, with a sharp decline 22 days before the event (12 days earlier than LAI and FPAR). The area was heavily vegetated with dominant landcover of forestry (26.4%) and improved grassland (23.1%), which may be more resilient to the hot and dry conditions and therefore less affected by lack of precipitation.

**Figure 11:**
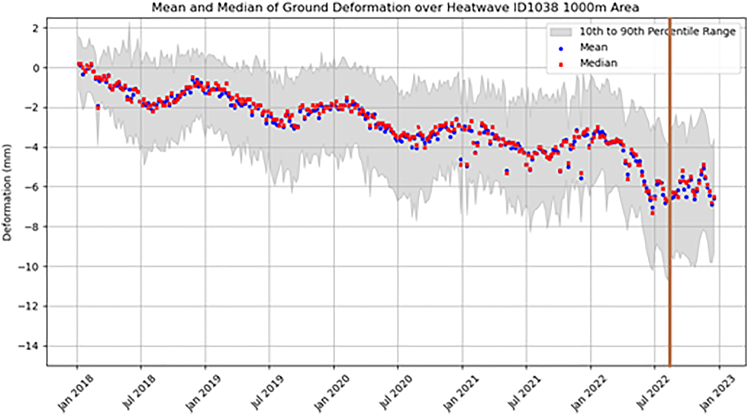
Time-series of average vertical velocity for a 1 km buffer surrounding Fordwich, associated with heatwave event on 11^th^ August 2022 Generated using European Union’s Copernicus Land Monitoring Service Calibrated 2019–2023 (vector), Europe, yearly data. DOI: https://doi.org/10.2909/8889e0a7-a6df-47a8-b4e7-a9cb32cbbf6b.

The ground motion of this area showed a general trend of subsidence (negative deformation) of ∼ –2 mm/year across the 2018–2022 period, noting all vertical motion was compared to the baseline at the start of 2018 (Figure 11). The trend showed a cyclical seasonality in deformation pattern seen as a downward trend (subsidence) observed in spring to early summer (February to June), followed by a period of stable motion in the summer months (July and August) before a period of uplift in the autumn to early winter (September to December) and stable motion throughout December and January. The heatwave event (indicated by the brown vertical line in Figure 11) showed an anomalous period of rapid subsidence (–4 mm) throughout the spring to early summer of 2022 followed by a relatively “noisy” period of uplift/subsidence with a 1 mm amplitude after the event. The observed ground motion was consistent with an area of clay and peat, particularly vulnerable to volume change with a reduction in moisture levels and resulting subsidence.

The event buffer lies on bedrock consisting predominantly of sand, silt and clay of the Thanet Formation (76.8%). The superficial deposits of this area are dominated by clay-rich alluvium with some peat (37.4%) and clay-rich head deposits (28.1%) together with river terrace sand and gravels (16.6%). The clay and peat components are particularly vulnerable to volume change as moisture levels reduce.

#### Pattern recognition for a specific wildfire event

**Figure 12:**
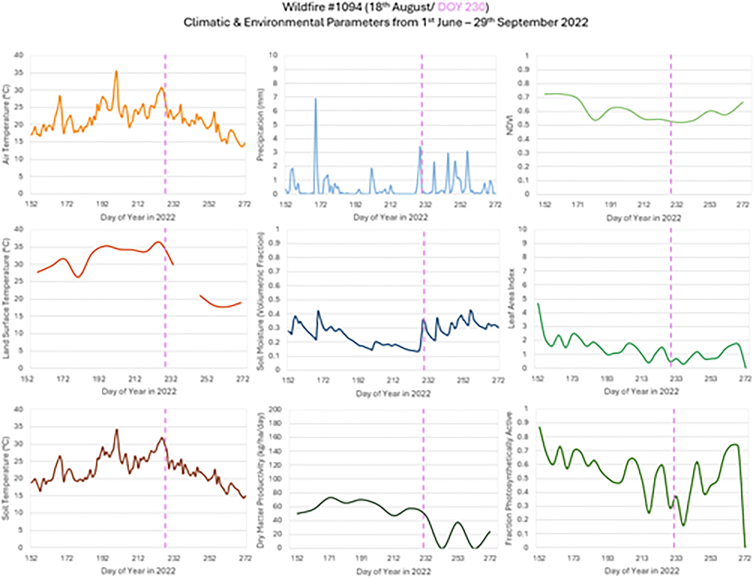
Time-series for climatic and environmental parameters for the period 1^st^ June–29^th^ September 2022 for a wildfire event in Kent (HIC event #1094) Local wildfire occurrence indicated with dashed vertical line. Contains data from modified Copernicus Climate Change Service Information (2022) ECMWF^50^ and Copernicus Land Monitoring Service products.

A wildfire occurred on Dartford Heath on 18^th^ August 2022 (HIC event #1094). This was the seventh event within a span of three weeks. Responded to by six fire engines, the grass fire spread across the heath due to tinder conditions. To prevent further fires over the following days, fire response services (FRSs) proceeded to dampen surrounding areas and hotspots. Climate variables and environmental parameters associated with this wildfire were examined for pattern recognition (Figure 12).

Air and soil temperature were consistently above the 17°C average from 6^th^ June, equating to 73 days of above average temperatures prior to the wildfire. Land surface temperatures showed a similar warming pattern, consistently 5°C above the air and soil temperatures and remained above 28°C for 45 days prior to the wildfire. During this time, precipitation remained low at 1 mm per day from 6^th^ June to 31^st^ August (53 days prior to up to 13 days after wildfire declared), with only three occurrences where there was a precipitation event greater than 1 mm/day before the wildfire event. Soil moisture gradually declined from 18^th^ June to 16^th^ August (61 days up to 2 days prior to wildfire declared). However, the trend showed step-like features during this time, which may be associated with the FRS dampening the heathlands to prevent fire hotspots due to the tinder conditions. As the air and soil temperatures declined from 24^th^ August (6 days after wildfire declared), both precipitation and soil moisture increased most likely due to the dampening of the heath. LAI and FPAR were at low levels and showed a shallow general decline from the beginning of the observation period. Both parameters experienced a sharp decline 25 days prior to the wildfire followed by gradual increase. There was a second sharp decline and more pronounced dip 5 days before the event followed by a sharp increase immediately before the wildfire. NDVI showed a similar pattern to the LAI and FPAR but much less pronounced decline of 0.2 and increase in NDVI. The increases in LAI, FPAR and NDVI may be linked to the vegetation response to the dampening of the heathlands which may allow the vegetation to begin to recover. DMP values declined from the beginning of the observation period with a sharp decline immediately after the event due to the lag in the time of recovery of vegetation. The sharpness of the vegetation parameter variations may also be attributed to the temporal resolution of the data as the health of surface vegetation can change rapidly over a 10-day period, particularly in wildfire prone areas.

The area within the event buffer is predominantly urban (43.4%) with a high cover of improved grassland (34%) and forest (18.2%); however, the area immediately surrounding the buffer is heavily vegetated with semi-natural grassland leading to the potential for wildfire if certain conditions were met.

**Figure 13:**
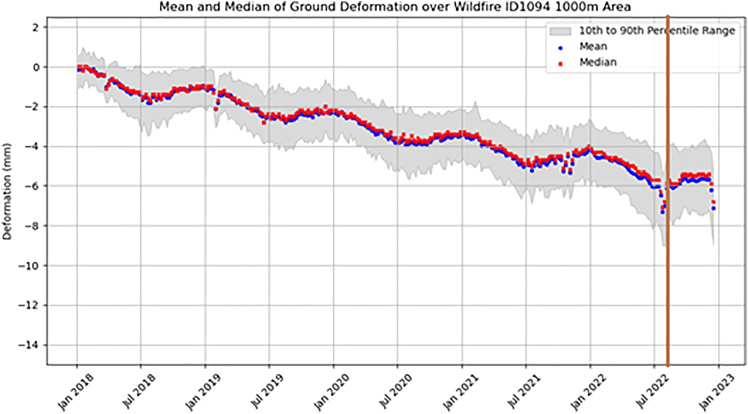
Time-series of average vertical velocity for a 1 km buffer associated with the wildfire event on 18^th^ August 2022 Generated using European Union’s Copernicus Land Monitoring Service Calibrated 2019–2023 (vector), Europe, yearly data. DOI: https://doi.org/10.2909/8889e0a7-a6df-47a8-b4e7-a9cb32cbbf6b.

The ground motion associated with the wildfire event was assessed by extracting all EGMS data points within a 1 km buffer around the event from the EGMS data (Figure 13). The graph indicates the vertical velocity for 203 points occurring in the 1 km buffer area associated with wildfire event. The graph showed a general trend of subsidence (negative deformation) of ∼ −1.75 mm/year across the 2018–2022 period, noting all vertical motion was compared to the baseline at the start of 2018. The trend showed a cyclical seasonality in deformation pattern with a downward trend (subsidence) observed throughout the spring to early summer (February to end of July), followed by a period of stable motion in the summer months (July to August) before a period of slight uplift (∼+0.5 mm) in the autumn months (September to November) and a stable period throughout December and January. The wildfire event (indicated by the brown vertical line in Figure 13) showed a pronounced short-lived anomalous period of subsidence (∼−1.75 mm) immediately before the event. While the wildfire and subsidence are not causally linked, they are considered compounding hazards, both influenced by the preconditions associated with the sustained high temperatures in southeast UK.

The event buffer lies on bedrock of sand of the Thanet formation (70.2%) and chalk of the Lewes Nodular, Seaford, and Newhaven chalk formations (29.8%). Superficial deposits within the buffer are dominated by sand and gravel of the Boyn Hill Terrace formation (71.1%) with the other superficial deposits containing clay (13.1%). Sand and gravel are not generally susceptible to climate-driven ground motions. However, the feature observed around the time of the wildfire may result from a change in coherence caused by surface alternations from the fire, with subsequent “noise” potentially due to increased moisture from dampening of the heathland.

#### Pattern recognition for a specific flash flood event

**Figure 14:**
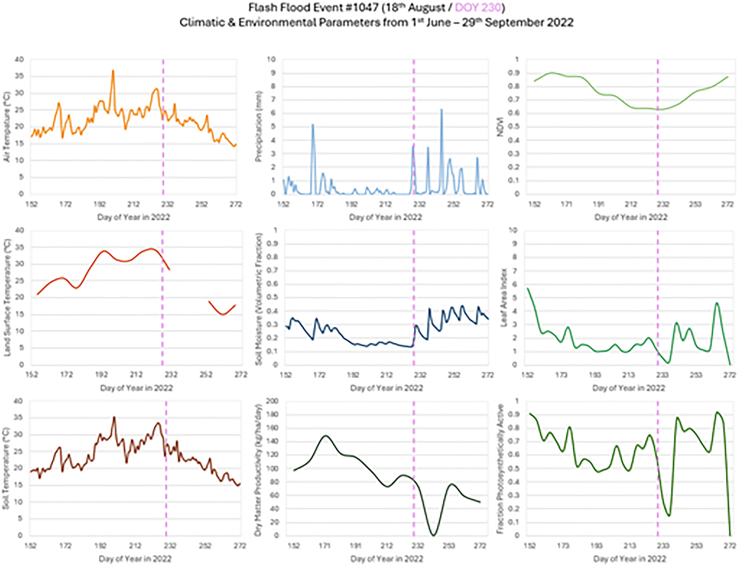
Time-series for climatic and environmental parameters for the period 1^st^ June–29^th^ September 2022 in Kent (HIC event #1047) Local flash flood occurrence indicated with dashed vertical line. Contains data from modified Copernicus Climate Change Service Information (2022) ECMWF^50^ and Copernicus Land Monitoring Service products.

A flash flooding event occurred in Frittenden on 18^th^ August 2022 (HIC event #1047) where it was reported that 46 mm of precipitation fell across the surrounding area within an hour. Travel disruptions were reported across local road and rail networks, with users advised to plan journeys ahead with caution. The area within the event buffer is predominantly improved grassland (72%) and arable (15.9%) with urban coverage (7.3%). Climate variables and environmental parameters associated with this flash flood event were examined (Figure 14).

Air and soil temperatures were consistently above the 17°C average from 30^th^ June and remained above 28°C (Met Office heatwave threshold for Kent) for 4 consecutive days prior to the event. However, both experienced a rapid drop immediately preceding the event on 18^th^ August attributed to an increase in thunderstorm activity. Land surface temperatures were consistently 5°C warmer than both air and soil temperatures during this time and followed a similar trend in warming pattern. Land surface temperature remained above 28°C for 45 consecutive days before the event. The slope of the decline in land surface temperature appeared less steep than that for air or soil temperature, but that may be an effect of the 8-day land surface temperature product and the timing of the relative observations. Precipitation remained low leading up to the event, with only one rainfall period on 18th June (61 days prior to the event), which matched the drop in air, soil and land surface temperatures at that time. Soil moisture levels also remained low from 5^th^ July due to the lack of precipitation through to the onset of the event between 16^th^ and 18^th^ August when soil moisture levels rapidly increased in conjunction with repeated strong precipitation events. LAI, FPAR and NDVI responded to the initial increase in temperatures and decrease in available moisture by a decline in value as a result of increased stress on the vegetation. The decline continued for 7 days after the event before LAI and FPAR values sharply increased 11 days after the event, with a lag in NDVI potentially due to the vegetation taking time to recover before regrowth. DMP declined significantly during the lead up to the event, as a response to the overall declining growth rate of the vegetation over this period. There was a sharp decline in DMP immediately after the event with a lag in regrowth rate post event before a further decline as the vegetation reaches maximum growth rate.

**Figure 15:**
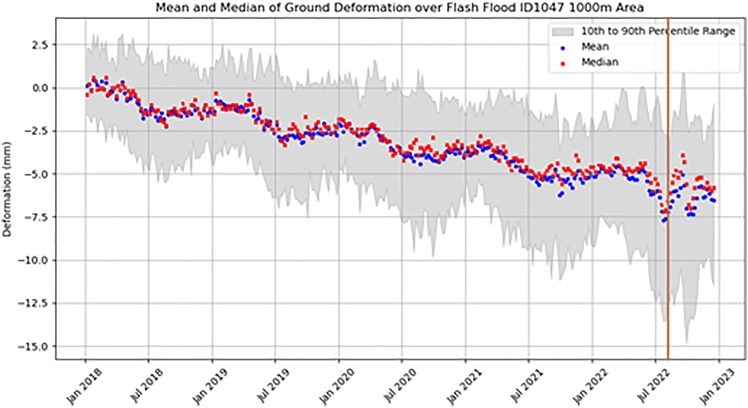
Time-series of average vertical velocity for a 1 km buffer associated with the flash flood event on 18^th^ August 2022 Generated using European Union’s Copernicus Land Monitoring Service Calibrated 2019–2023 (vector), Europe, yearly data. DOI: https://doi.org/10.2909/8889e0a7-a6df-47a8-b4e7-a9cb32cbbf6b.

The ground motion associated with the flash flood event was assessed by extracting all EGMS data points within a 1 km buffer around the event from the EGMS data (Figure 15). The graph indicates the vertical velocity for 203 points occurring in the 1 km buffer area associated with flash flood event. The graph showed a general trend of subsidence (negative deformation) of ∼ −1.7 mm/year across the 2018–2022 period, noting that all vertical motion was compared to the baseline at the start of 2018. The trend showed a cyclical seasonality in deformation pattern with a pronounced downward trend (subsidence) of ∼ -2 mm in the spring to early summer (February to July), followed by a period of minor uplift (∼+1 mm) throughout August to February. The flash flood event (indicated by the brown vertical line in Figure 15) corresponded to an anomalous period of subsidence (∼-3 mm) in the month immediately before the event and was followed by a “noisy” period of ground motion to the end of 2022.

**Figure 16:**
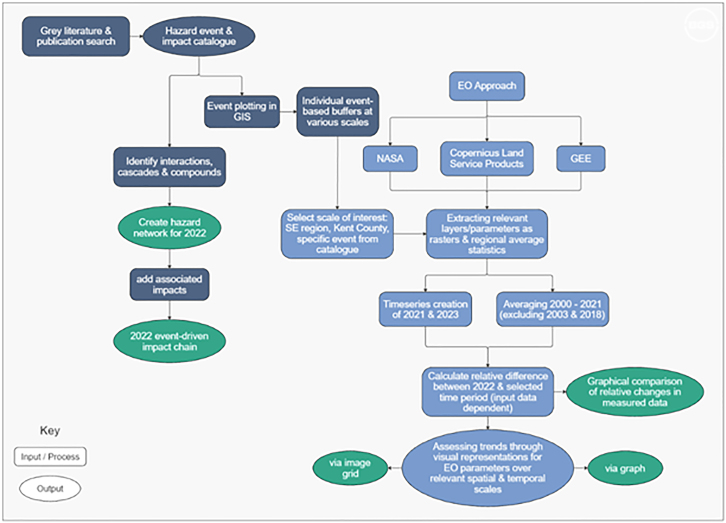
Flow diagram representing an overview of the key methodological elements for the southeast UK study

**Figure 17:**
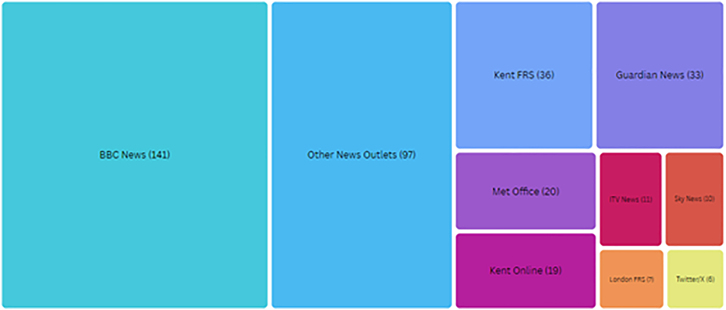
Tree map capturing the gray literature used to complete the internal multi-hazard catalog specific to events across the UK from early 2018 to late 2023

**Figure 18:**
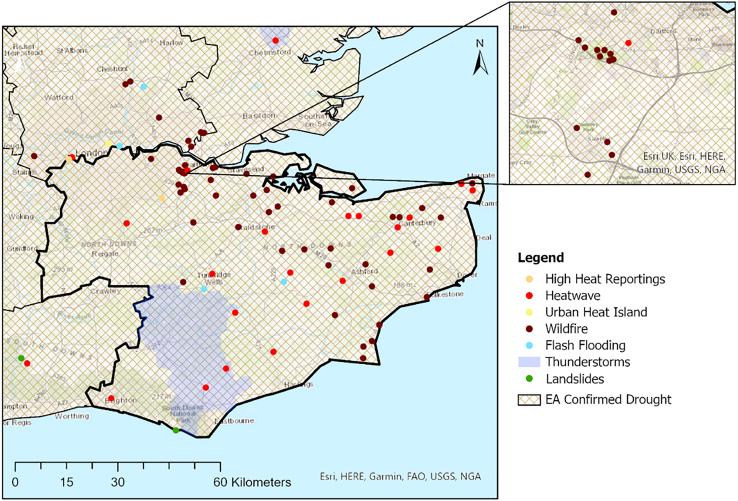
Reported hazards and impact events across southeast England, from 1 June 2022 to 31 August 2022, collated and mapped from the hazard impact catalog through GIS Events are plotted on the topographic basemap layer in ArcGIS Pro (Source: Esri UK, Esri, HERE, Garmin, FAO, USGS, and NGA).

**Figure 19:**
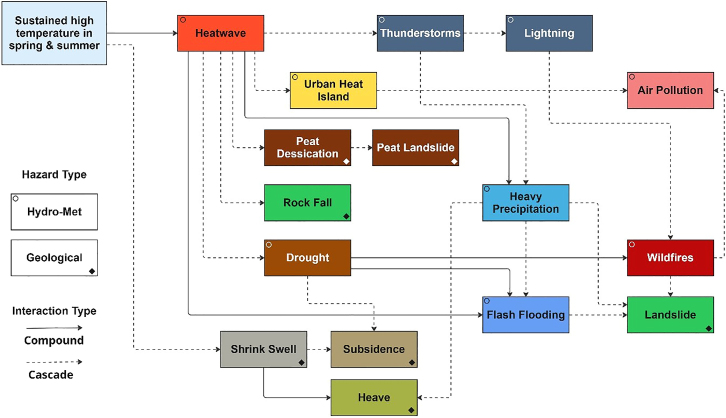
Hazard chain for southeast UK detailing cascading and compounding climatic and geological hazards between early June and late August 2022

**Figure 20:**
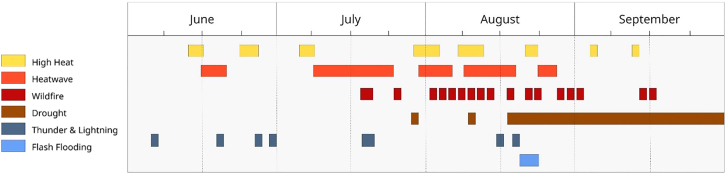
Temporal chain of hazard events from June to September 2022 in Kent, constructed from the hazard impact catalog

**Figure 21:**
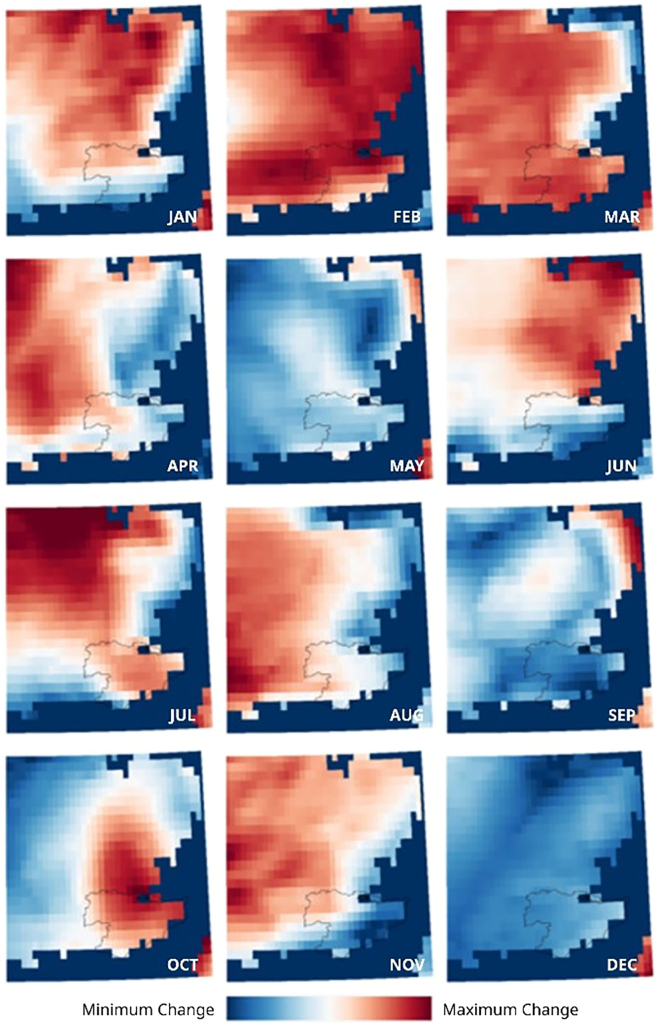
Temperature difference in ECMWF monthly maximum air temperature for southeast UK calculated as the difference between 2022 and the average from 2000 to 2021 (excluding 2003 and 2018 as known anomalous years) for each month The color represents the minimum (blue) to maximum (red) temperature change for any given month highlighting spatial distribution patterns. Contains modified Copernicus Climate Change Service Information (2022) ECMWF data.^50^

The event buffer lies on a bedrock of mudstone (67.5%) and limestone (27%) of the Wealden group. Superficial deposits within the buffer are sparse with the presence of clay-containing river terrace deposits (7%) and alluvium (3.5%). Extending from this buffer zone, Frittenden sits on the Weald Clay that is of medium plasticity with shrinking properties exacerbated by the geographical location in conjunction with climate. Although the flash flood resulted from high precipitation, the preceding high heat caused a hardening of the ground so it is possible that the induration and ground drying also led to the observed ground motion pattern.

## Discussion

The compounding effects of repeated atypical climate conditions, along with the resulting hazards and impacts, provide a strong basis for investigating hydrological and meteorological droughts in the UK context. By analyzing precondition signatures and patterns in EO data for specific localized events, it is possible to identify initial environmental and climatic commonalities among hazard events across space and time. This descriptive study specifically compared the similarities among multi-hazard events associated with the 2022 period of sustained high-heat temperatures, in relation to known impacts. All anomaly and threshold observations are retrospective and descriptive. Associations between indicators and events should not be interpreted causally; they motivate testable hypotheses for future validation.

The detail from the pre-conditioning, baseline and observed compounding conditions for *heatwave events* across southeast UK, suggested that there were at least 55 consecutive days with air temperatures above 15°C, followed by 10–25 days of temperatures between 19°C and 22°C. These were often compounded by temperature spikes in the days prior to event onset. Patterns for soil temperatures and LST follow a similar trend with an average of 30 consecutive days at temperatures above 16°C and 23°C, respectively, compounded by between 20 and 40 days above 22°C and 27°C, again peaking just prior to event onset. Soil moistures subsequently declined, with consecutive values of below 0.3 in the 50 days prior to event onset. The vegetation parameters showed an inverse pattern in relation to the temperature and moisture-related parameters, similar to patterns documented associated with heatwaves.^53^^,^^54^^,^^55^

*Wildfire events* pre-conditioning across the county of Kent events highlights baseline air temperatures above 16.5°C for at least 45 consecutive days, increasing toward the event onset and compounded by 3–5 days of 26°C just prior. Precipitation levels show that at least 30 days with below 3 mm recorded, compounded by 10 days of below 0.5 mm with little to no precipitation recorded in the few days prior to event onset. The environmental parameters highlight the vegetative response prior to event onset, with an average of 50 days with NDVI above 0.5 and steady increase in LAI and FPAR leading up to the event, with significant drops in the immediate aftermath. Dry matter productivity showed the same trend as increase in air temperatures and a lack of precipitation, with 45–70 days of 40–160 kg/ha/day prior to event onset and declined after the event. These observed patterns were consisted with patterns documented in relation to wildfires.^56^

*Flash flooding* that occurred in the county of Kent had varying pre-conditioning and baseline air and soil temperatures. The flash flood was compounded by a minimum of 20 days of air temperatures of at least 20°C and over 20 days of soil temperatures above 21.5°C prior to the event. Soil moisture was seen to decline pre-event and increased in a lagged response after the event onset, linked with increased runoff and decreased infiltration associated with drying. There moisture levels remained below 0.4 for between 50 and 100 days prior to the event, compounded by 10–20 days with levels below 0.2. The pre-conditioning precipitation across events remained below 1.5 mm for 30–50 days, followed by 25–30 days of less than 0.5 mm, and finally compounded by 16 days of less than 0.01 mm recorded across the area just prior to onset.

While these preconditioning signals and patterns are presented descriptive, we acknowledge that the available observations were insufficient to support statistically robust validation or confidence-interval estimation. Future work could therefore explore formal anomaly testing and threshold evaluation where suitable, independently verifiable datasets (e.g., published health statistics or high-resolution insurance records) become available.

Further, the present analysis characterizes associations between EO-derived indicators and recorded events after the fact. By design, it does not estimate statistical performance metrics (e.g., precision/recall, false-alarm rates, etc.), quantify lead times, or implement automated detection. In contrast, an operational early-warning system would require, among other: (i) independently verifiable impact datasets at appropriate spatial and temporal granularity; (ii) pre-registered detection and threshold rules; (iii) out-of-sample performance assessment with confidence intervals; (iv) evaluation of actionable lead times; and (v) an engineered, automated data pipeline with stakeholder-agreed activation protocols and governance.^57^^,^^58^ These elements fall outside the scope of the current, post-event study, which is intended to provide a transparent evidential foundation upon which such developments could later be built.

### Comparison of study results with *in situ* climate assessment from HadUK

A review of the UK climate and environmental data along with the impact chain analysis confirmed that the 2022 heatwave was an anomalous climate event that led to several further hazards and impacts that are rarely seen in the UK. The EO-based assessment related to the time-period of 2000–2022 due to data availability and the trends were compared to *in situ* data from HadUK (https://www.metoffice.gov.uk/research/climate/maps-and-data/data/haduk-grid/datasets), a long-term climate gridded dataset produced by the Met Office that covers time-periods 1961–1990 and 1991–2020, providing monthly, seasonal, and annual averages of climate variables.

Overall, the UK mean temperature for 2022 was 10.0°C, 0.9°C above the 1991–2020 long term average and the warmest year on record across all 4 nations since records began in 1884. The highest anomalies relative to 1991–2020 were across central and eastern England (+1.0°C), and the lowest across northern Ireland (+0.7°C). The UK mean temperature was 1.7°C above the 1961–1990 and 1991–2020 long-term average.^30^

This pattern of warming was observed where the most recent decade 2013–2022 was 0.3°C warmer than 1991–2020°C and 1.1°C warmer than 1961–1990. Significantly more warming occurred across England and Wales, and slightly less across Scotland and Northern Ireland. The UK annual mean daily maximum temperature (Tmax) for 2022 was 13.9°C, 1.1°C above the 1991–2020 long-term average and the warmest year in the UK Tmax series from 1884 by a considerable margin, with the next warmest daily average in 2014 at 13.5°C. The UK annual mean daily minimum temperature (Tmin) for 2022 was 6.2°C, which is 0.6°C above average, with anomalies generally lowest in the south.

All months of the year except December were warmer than the 1991–2020 average. Anomalies were +1.0°C or higher in 7/12 months (February, March, May, July, August, October, and November). August 2022 was the equal-third warmest august for Tmax and has only been two calendar months warmer than August 2022 in the last 10 years (July 2018 and July 2013).

In addition to anomalously high temperature, the precipitation data reveals that the UK received 6% below average precipitation (1991–2020). Despite this, there has also been a slight increase in heavy precipitation in recent decades, which may explain the increased incidence of flash flooding events.^59^ There is also evidence that sea level around the UK has risen by 18.5 cm since 1900s, with the rate is increasing with over 60% of the (11.4 cm) occurring over the past 30 years,^60^ highlighting a future research avenue with respective hazard-EO integration.

### Limitations of the study

The aim of this case study was to assess the potential of EO data to identify candidate thresholds leading to sustained high-heat events and to monitor these events once they occur. With climate change expected to increase the frequency and severity of such events in the UK,^61^ leveraging EO-derived products is essential for supporting decision-making and reducing risks associated with multi-hazards to key sectors and stakeholders. In this study we assessed, the potential use of EO-derived data for monitoring sustained high-heat events in the UK. While this method is based on the HIC and is more applicable to areas where a lot of information is available about a hazard event—where the level of detail about the event is essential—it is less applicable where similar supporting data are unavailable. Therefore, if no detailed understanding of the event is forthcoming through the different sources, then it becomes difficult to identify plausible trends and assess the event within space and time. Additionally, while trends in EO-derived parameters were identified, current temporal and spatial resolution are insufficient for effective monitoring at a regional scale. Indeed, this objective is hindered by the following limitations:

(1) Event geolocation and buffer zones. The geolocation of the hazard and definition of the buffer zones are defined with an approximate position based on the evidence extracted from the gray literature, in conjunction with contextual landscape information when defining the position of the event within the hazard impact catalog (HIC). This has limited the precision at which hazard-specific analyses can be conducted, particularly for small-scale or localized events, and introduces uncertainty when linking environmental and climatic patterns to observed impacts.
(2) Spatial and temporal resolution: to effectively support local decision-making, EO data must be available at high spatial resolution and with frequent temporal coverage to measure and derive all relevant parameters. This would enable rapid assessments and the near real-time monitoring essential for early warning efforts. Currently, however, key satellite repeat times are not frequent enough to support this type of data interrogation and the data returned is also often at a resolution that is not compatible with local assessments such as for flood mapping.^62^^,^^63^^,^^64^
(3) Cloud-cover limitations: satellite observations used in the above analyses measure within optical wavelengths, which are opaque to cloud cover. In this study case, we commonly have cloud cover during our observational period which limits the opportunities for direct observation of the ground surface and therefore hinders generation of associated derived parameters (such as LST, LAI, FPAR, and/or NDVI). While satellite-based Sentinel-1 radar data can penetrate cloud cover with a relatively high spatial resolution, its application is limited when it comes to directly extracting land surface temperature (proxy from soil moisture) or detailed vegetation characteristics. Radar-derived metrics are generally restricted to simple vegetation indices^65^ or used for monitoring phenology and crop development,^66^^,^^67^^,^^68^ rather than providing the detailed vegetation parameters explored in the case study.
(4) Event detectability: spatial resolution of the satellite observations used in the above analyses are relatively coarse (e.g., daily climatic data from ECMWF at 11 km (resampled to 1 km), or 12-hourly temperature data from MODIS at 1 km) for observing events that occur over a small spatial extent. For example, if there is a small wildfire that covers only a small fraction of a pixel, the wildfire algorithm does not identify the associated wildfire and therefore as a result, only the larger wildfires in the event catalog are observed.
(5) Ground motion monitoring constraints: data such as that provided by the European Ground Motion Service (EGMS) is typically released annually, packaged into five-year intervals that reset to a new baseline every five years. This approach, while useful for detecting short-term ground motion trends, poses challenges for observing and interpreting longer-term changes, particularly at the transition between intervals. The periodic baseline resets can obscure continuity, making it difficult to distinguish between transient phenomena and gradual, cumulative trends that span beyond the five-year windows. As a result, analysts may face limitations in reliably assessing long-term ground deformation patterns or in comparing trends across multiple observation periods. In addition, the EGMS covers only Europe so adapting this work to other global regions will be more challenging and require additional resources to replicate the EGMS service to other geographic regions.
(6) Data processing, infrastructure, and capacity development: the rapid assimilation of large satellite datasets requires investment in both computing infrastructure and staff expertise. While much of the data are accessible through Google Earth Engine (GEE) and coding has been designed to extract specific parameters from specific image collections appropriate to the case study, not all data are available through this portal and therefore access to multiple data portals is required. Additional reprojection of datasets or conversion of the time element (comparison between daily, 8-day or 16-day) is required before direct comparative analysis can be performed. The technical and infrastructural challenges associated with processing large satellite datasets also pose limitations. The need for investment in computing infrastructure and staff capacity development is critical, as is ensuring that data from multiple platforms can be accessed and integrated effectively for comprehensive analysis. The existing issues with data accessibility, reformatting, and comparison of datasets hinder the timely processing of EO data, which is crucial for emergency response decision-making.
(7) Climatic and socio-economic context: while this case study is focused on the unfolding of the 2022 event in southeast England, it limits the hazards and associated impacts to this specific context. Expanding this work to other contexts to include other hazard compounds and cascades, and complex societal, environmental and economic impact interactions would highlight the frameworks applicability. The method is driven by the availability of impact data stemming from real world hazard events, which may be difficult when transferring the framework to data sparse areas. While the socio-economic component is not a focal point of this study, it provides a basis for future work—particularly regarding dynamic exposure and vulnerability to a hazard event.
(8) Conceptual scope and transferability: while this study focuses on a single, well-documented event in southeast England, the workflow has been designed to be transparent and replicable rather than prescriptive or universally transferable. The integration of EO-derived indicators with empirically constructed impact chains reflects the unique availability, resolution, and coherence of data for the 2022 high-heat episode. Accordingly, the findings or candidate thresholds cannot directly be generalized across different climatic, geological, or socio-economic contexts. Extending the workflow to other regions or time periods would require hazard reporting, EO observations, and impact datasets of comparable granularity and quality, which currently vary widely across the UK and internationally and can form the object of future work.

### Concluding remarks

The 2022 heatwave in the UK was an exceptional climate event, with the national mean temperature reaching 10.0°C, marking the warmest year on record. This year surpassed previous temperature averages by 0.9°C, with central and eastern England experiencing more pronounced warming. The temperature trends from 2000 to 2022 demonstrated a clear pattern of warming, with the most significant increase in temperature observed across England and Wales. The UK also experienced high maximum and minimum temperatures in 2022, with several months exhibiting anomalies above 1.0°C. While precipitation decreased by 6% compared to the long-term average, there has been an increase in heavy precipitation events, which has contributed to a rise in flash flooding occurrences. Additionally, sea levels around the UK have risen by 18.5 cm since the 1900s, with the rate of increase accelerating in recent decades, highlighting the ongoing environmental changes that are impacting the country.

This study demonstrates the potential of EO data to identify candidate thresholds and patterns associated with sustained high-heat events and related hazards. By integrating climate variables, environmental parameters, and ground motion data within the developed framework of data-driven impact chains, we were able to reveal preconditioning factors and emerging trends across space and time. The analysis of specific events in Kent illustrated how multi-hazard cascading and compounding can occur under shared preconditions, such as prolonged high temperatures, highlighting the value of EO-derived parameters for supporting future multi-hazard assessment and early warning potential.

The protocol developed and subsequently used throughout this study highlighted the application of EO-derived data for environmental and climatic thresholding of small-scale hazard events in the UK. When used in combination with products such as EGMS, corresponding ground responses to these multi-hazards can be observed in relation to known bedrock and superficial geologies. In areas with deposits of a high clay content, ground motion patterns highlighted anomalies throughout the summer months of 2022, in addition to seasonal cycles and general deformation trends over time. This suggested a high susceptibility of the clay-rich deposits to heat-driven ground motion.

The trends and candidate thresholds identified in this study provide a foundation for developing tools and methods for integrating EO data, its derived parameters and impact data via empirically driven impact chains, to align with semi-automated monitoring and early warning potential of multi-hazard events. The development of these tools, methods and capabilities can be iteratively improved in consultation with stakeholders, ensuring that the proposed protocol can be developed to operationalize and support proactive multi-hazard detection.

However, several limitations constrain the full operational use of EO data for regional-scale monitoring. The geolocation of hazards and buffer definitions, derived from gray literature and contextual landscape information, limits the precision of event-specific analyses. The spatial and temporal resolution of current EO datasets remains insufficient for capturing small-scale or short-lived events, while cloud cover restricts the utility of optical satellites for continuous observation. Radar-based datasets, such as Sentinel-1, offer cloud penetration but are limited in capturing detailed vegetation and land surface temperature characteristics. Similarly, the periodic release and baseline resets of ground motion data from the European Ground Motion Service complicate the analysis of long-term trends. Technical and infrastructural challenges, including the assimilation and harmonization of large multi-source datasets, further limit timely operational application.

Identifying trends, preconditioning factors, and potential thresholds paves the way for near real-time hazard detection and supports informed decision-making to mitigate future risks. Future work should focus on improving spatial and temporal resolution, enhancing radar-based parameter extraction, advancing and refining algorithms for detecting localized hazards with means of validation, and strengthening data-processing infrastructure. Building on the results in this study, further work will focus on the relationship between the clay-rich deposits and climate and environmental conditions, using BGS Property Subsidence Assessment (PSA) (https://www.bgs.ac.uk/datasets/property-subsidence-assessment/) and BGS GeoSure (https://www.bgs.ac.uk/datasets/geosure/), and the impact of sustained high-heat events on exposure and vulnerability across different sectors.

Our approach is intentionally pragmatic: we synthesize established EO products within a structured, testable workflow. Rather than proposing a new algorithm, the contribution lies in delivering a transparent, end-to-end framework—from HIC development and impact chain encoding to indicator screening, anomaly assessment, and candidate threshold identification—explicitly designed for replication and prospective validation. The study underscores the value of EO datasets to the retrospective analysis and monitoring of high-heat multi-hazard events in the UK, a region where such studies remain relatively limited, offering candidate indicators for future validation rather than an operational early-warning system. By integrating observational data with empirically derived impact chains, the framework demonstrates promise in detecting early signals of hazard preconditions and revealing linkages among climatic and environmental parameters associated with cascading and compounding hazards. Collectively, these results highlight its potential to support more proactive multi-risk assessment and management in the context of a warming climate.

## Resource availability

### Lead contact

Requests for further information and resources should be directed to and will be fulfilled by the lead contact, Erin Mills (emills@bgs.ac.uk).

### Materials availability

All unique results generated and subsequent code used in this study are available to request from the lead contact with a completed materials transfer agreement with the caveat of not for commercial use.

### Data and code availability

• This article analyses existing, publicly available data. The data source can be shared by the lead contact upon request.
• The article does not report original code, and scripts used can be shared upon request.
• The hazard impact catalog is currently held within the organization, and excerpts can be made available upon request subject to intended use.
• Any additional information required to reanalyze the data reported in this article is available from the lead contact upon request.

## Acknowledgments

This research was supported by the European Space Agency (ESA) under the EO4MULTIHA project (2023–2025), contract number 4000141754/23/I-DT.

## Author contributions

Conceptualization, R.C. and A.W.; methodology, E.M. and K.S.; data collection, E.M. and K.S.; writing – original draft, E.M. and K.S.; writing – review and edits, R.C., A.W., and L.B.

## Declaration of interests

The authors declare no competing interests.

## STAR★Methods

### Key resources table

**Table:** 

REAGENT or RESOURCE	SOURCE	IDENTIFIER
**Deposited data**		

Earth Observation data used throughout this study	Detailed in the supplemental information	N/A
Hazard-Impact Catalog data used in this study	Excerpt detailed in supplemental information	N/A
Candidate threshold values for specific hazard events mentioned in this study	Detailed in the supplemental information	N/A

**Software and algorithms**		

Google Earth Engine	Google Earth	https://earthengine.google.com/
ArcGIS Pro 3.4.0	Esri Inc	https://www.esri.com/en-us/arcgis/products/arcgis-pro/overview
Miro Board	Miro	https://miro.com/index/

### Method details

The objective of the study was to investigate the complex and interacting nature of multiple hazards related to sustained high-heat, namely heatwaves, drought, wildfires, thunderstorms, flash flooding, landslides, and subsidence occurring in 2022. To achieve this, the study compiled evidence from official and gray literature sources to build a Hazard Impact Catalog (HIC). This catalog was used to examine the relationships between hazard events, focusing on whether they occurred as compounding or cascading events. The timing, location and severity of these hazards were then analyzed in relation to spatiotemporal patterns derived from earth observation data and associated climate and environmental parameters.

The methodology for multi-hazard analysis highlighted in Figure 16, shows both the data collection from literature and EO approaches, and how the processing and outputs of the two are integrated. The Hazard Impact Catalog forms the basis of the methodology, with climatic and environmental parameters extracted for locations specific to where hazardous events were reported and associated impacts have occurred.

#### Development of Hazard Impact Catalog (HIC)

The Hazard Impact Catalog (HIC) was designed to gather impact-driven information, documenting the type of hazard together with detailed information on its characteristics and impacts across different sectors. This approach enables a comprehensive understanding of how hazards interact across space and time, facilitating the identification of patterns and interdependencies.

This case study is evidenced by an extensive catalog of multi-hazard events and the associated impacts from periods of sustained high-heat across the UK from 2018 to 2023. The events were accumulated from over 380 open-source publications and gray literature. These include UK-based news reports, policy briefings government and or public sector reporting (Figure 17). From this dataset, 210 reports specifically relate to the heatwave cascades from early June to late August 2022, with a spatial focus on southeast England. This dense data cluster forms the basis of the EO data and impact chain analyses and is fed into the ESA EO4Multihazards online events database (https://eo4multihazards.eurac.edu/).

The hazard and impact records were accessed via Google research engine, and the data collection began as the event was unfolding in the summer of 2022. During the development of the HIC, little to no peer-reviewed data was available to assess prior to the events. However, timely peer-reviewed articles were subsequently published and those are discussed in relation to the impact chain analysis later in this publication. The following criteria were applied when sourcing information for inclusion in the HIC.

• **Time interval**: While the primary focus of this study was the sustained high-heat events of 2022, the analysis was extended to include other significant high-heat events within the region to better understand multi-hazard dynamics. The last comparable event occurred in the summer of 2018; therefore, the Hazard Impact Catalog spans from February 2018 to December 2023. This temporal window was selected to capture potential antecedent conditions influencing the severity of the 2022 events, as well as to account for delayed or cascading impacts that continued up to 18 months beyond the main heatwave period.
• **Search terms**: The keywords used to identify relevant articles were: ‘high-heat’, ‘heatwave’, ‘wildfire’, ‘grassfire’, ‘air pollution’, ‘flash flooding’, ‘thunderstorm’, ‘drought’, ‘rockfall’, ‘landslip’, ‘landslide’, ‘subsidence’, ‘heat alert’, ‘heat warning’, ‘urban heat island’. These keywords were used in Boolean search strings (e.g., using “AND”/“OR” operators) in combination with temporal and spatial filters such as date ranges and specific UK locations (e.g., Kent, East Sussex, Greater London).

Anything falling within the above criteria was recorded in the catalog, given a geolocation (corresponding to most appropriate position based on the documented detail and landscape context) and given a unique reference number (identified as #YYYY). The information captured in the HIC detailed whether the event was a primary or secondary event (directly related to a preceding event), date, duration, spatial extent and/or magnitude of event and number of fatalities. Additionally, it documents the use and specifics of any satellite imagery and/or data used, the stakeholders affected or involved with the event response, the nature of the impacts and all sources of information.

Using the information recorded throughout the HIC, events that occurred across the southeast were spatially plotted in a Geographic Information System (GIS) to produce a geospatial representation of the catalog. Each individual hazard event occurring between the beginning of June and the end of August 2022 was attributed with geospatial coordinates, representing a point location, with attributes given to emulate the data contained within the catalog. Where possible, the spatial extent of the event was represented or acknowledged based on the information drawn from each catalog source, forming the basis for the EO data analysis. Using the Geoprocessing Tools within the GIS, a series of buffers were produced at various extents around each event in the HIC (radius: 10m, 100m, 300m, 500m, 1 km or 10 km), representing the most appropriate spatial scale of the event (if available) and exported as shapefiles for use in collating relevant EO and environmental data for time-series analyses. It is worth noting that the area of potential impact is not constrained to this spatial buffer. An overview of the data plotted within GIS is shown in Figure 18.

The comprehensive information within HIC facilitated the development of a multi-hazard impact chain, providing a visual tool to better understand the complex hazard interactions in southeast UK during 2022.

#### Development of multi-hazard impact chains

An impact chain is traditionally a conceptual model that visually represents complex cause-effect relationships in a simplified manner, making them more accessible to stakeholders and non-scientists. While first developed for climate risks,^69^ impact chains offer a structured method to capture the most relevant factors contributing to a specific risk.^70^ These factors include the hazards and their cascading effects (impacts), exposure, and vulnerability, which together determine the manifestation of risk in a particular context (e.g., regional or sectoral). Impact chains are a recent - often hypothetical - application to highlight multi-hazard interactions and the resultant impacts across various sectors.^71^ Enabling a clear visual understanding of the hazard interactions, the resource can be integrated into mitigation and planning for future hazardous events and cascades – as this approach may benefit future risk assessments and the identification of critical paths for intervention and adaptation. In this study, the hazard impact chain is empirically-driven and constructed using the HIC, which provides information on recorded hazard events with the most accurate approximation of spatial and temporal distributions based on detail from the literature sources. In this study, hazard that coincide both spatially and temporally are considered compounding hazards, while hazards that overlap spatially but occur at different times are treated as cascading hazards. Using the events recorded in the HIC, data-driven impact chains have been produced to represent the hazard-impact relationships across southeast UK.

Initially, a generalised impact chain was created for all recorded events related to the 2022 sustained high-heat event and information was added about the precursory conditions that may have had an impact on the hazard cascade. The relationship between hazards was displayed graphically as a network diagram (Figure 19). This was split into physical hazards and their resultant impacts, grouped by sector: transport, public health, ecosystems, domestic, industry and water management. From here, all the branches of the network that relate to southeast UK were identified and a subset of the catalog was created to represent this data.

Building on the general impact chain, a temporal chain is derived to capture how hazards unfold and interact over time. By sequencing events along a timeline, it becomes possible to trace when and how different hazards overlap or follow one another, thereby identifying potential compounding or cascading dynamics in relation to the HIC (Figure 20).

In developing the HIC, locations were identified as potential case studies for integrating exposure and vulnerability into a multi-risk analysis. This aspect will be explored further in a separate part of this study and will not be addressed here. Since the conclusion of the southeast UK study, the catalog has been extended to include information on sustained high-heat events across Europe and globally, providing a broader context to the southeast UK events and will be examined in future research.

#### Earth observation (EO) data and derived parameters

A central question of this study is whether satellite-based EO data and their derived parameters can aid in identifying trends and tipping points in environmental and climatic variables that cause and or exacerbate both cascading and or amplifying effects in a multi-hazard event. To address this, a hazard impact chain was designed to assess the interrelationships between hazards captured in the HIC. The resulting information was used to link the timing, location, and severity of the hazards, enabling the assessment of spatiotemporal patterns and potential trigger points of environmental and climatic variables observed in EO data and their derived parameters.

There are two main categories of EO data considered this study for assessing potential indicators of trigger points in hazard assessment: climate and land surface. Selecting the most appropriate data requires consideration of several factors: (i) the temporal coverage (i.e., 2000-present), (ii) temporal repeat (i.e., 1-day, 4-day, 8-day, 10-day, 16-day, 1-month), (iii) low likelihood of presence of cloud within products (i.e., cloud-masked), and (iv) spatial ground sample distance (i.e., 10m, 100m, 300m, 500m, 1 km, 10 km) in relation to the events documented in the HIC, and (v) the environmental parameters that can be derived from the data. While the study aimed to use the most appropriate cloud-free products, it was inevitable that cloud-presence limited the completeness of some dataset collections, with others having a restricted temporal range and/or coarser spatial resolution than desired for event-level assessment.

Appropriate climate data were selected from the ERA-5 Land ECMWF reanalysis daily and monthly aggregate datasets,^50^ in particular focusing on the variables associated with maximum air temperature (K), maximum accumulated precipitation (m), maximum soil temperature (K) of the upper layer (0-7 cm soil depth), maximum volumetric soil moisture (fraction) of the upper layer, and surface runoff (m). ECMWF data was accessed using the Google Earth Engine (GEE) cloud computing platform.

Land surface derived environmental data products were selected as land surface temperature, (LST, K), fraction of photosynthetically active radiation, (FPAR, %), leaf area index (LAI, fraction) and the daily fire mask from NASA Aqua/Terra MODIS accessed using the GEE open-access online portal, with normalised difference vegetation index (NDVI, fraction) and dry matter productivity (DMP, kg/ha/day) from the Copernicus Land Monitoring Service (CLMS) (https://land.copernicus.eu/en).

In addition to climate variables and environmental parameters, geospatial county boundaries were obtained from the EA, and the wider hazard context was assessed using geological information from the British Geological Survey (BGS) Digital Geological Map of Great Britain project (formerly known as DiGMapGB) (https://www.bgs.ac.uk/datasets/bgs-geology-50k-digmapgb/) Bedrock Geology and Superficial Geology layers, alongside surface land cover derived from the aggregated 2022 UK Center for Ecology and Hydrology (UKCEH) land cover map (Aggregation from the original 21 UKCEH Land Cover classes and data for 2022 as: Forest (Broadleaf: class 1, and Coniferous Woodland: class 2); Arable (class 3); Improved Grassland (class 4); Semi-Natural Grassland (Neutral Grassland: class 5, Calcareous Grassland: class 6; Acid Grassland: class 7; Fen Marsh and Swamp: class 8, Heather: class 9; Heather Grassland: class 10; Bog: class 11; and Inland rock: class 12); Water/Coast (Saltwater: class 13, Freshwater: class 14; Supralittoral rock: classes 15; Supralittoral sediment: class 16; Littoral rock; class 17; Littoral sediment: class 18; Saltmarsh: class 19); and Urban (Urban: class 20, and Suburban: class 21). Full detail on UKCEH Land Cover^72^). Processed vertical ground displacement (mm) from Sentinel-1 was also extracted from the European Ground Motion Service (EGMS) (https://land.copernicus.eu/en/products/european-ground-motion-service) to examine the relationship between time-series trends in vertical ground motion and periods of sustained high temperatures, as well as heatwave-associated hazards.

#### Analysis of EO data in relation to the 2022 sustained high-heat event

Data associated with each climate variable and environmental parameter were extracted from the appropriate source for 2000–2023 (or as far back toward 2000 as possible) for regions of interest defined at 3 different spatial extents: (i) for the whole of the southeast UK; (ii) for the county of Kent; and (iii) for buffer zones defined with a radius of 10m, 100m, 300m, 500m, 1 km and 10 km centered around each 2022 event within the HIC.

To investigate the 2022 sustained high-heat event, geospatial time-series statistics and images were extracted for each of the daily and monthly aggregate climate data variables and environmental parameters at two spatial extents: (i) southeast UK; and (ii) the county of Kent, across the 2000–2023 period, using code developed in GEE. To ensure consistency with the units recorded in the HIC, temperature variables were converted from K to °C, with precipitation and runoff variable units converted from m to mm within the extraction code.

The code generated a time-series multi-band image stack, each layer representing a geospatial image for a day or month, depending on the input dataset. These layers were extracted chronologically for each climate variable or environmental parameter across two spatial extents: (i) southeast UK; and (ii) the county of Kent. Data were extracted for each year across the 2000–2023 period, noting that the derived parameters from the CLMS (Sentinel-2) were available for only 2021–2023 during the study.

The average for each day or each month was calculated across 2000–2021, excluding 2003 and 2018 as these were known to be anomalous years. A comparison was then performed by subtracting the 2000–2021 average from 2022. The resultant data provided an indication of the change in temperature each time slice in the image stack. A visual geospatial representation of the sustained high-heat event for southeast UK is shown in Figure 21 using the monthly data where the absolute temperature differences between 2022 and 2000–2021 average is displayed with a color ramp stretched for each individual image between the minimum temperature difference (blue) and maximum temperature difference (red). As the color stretch in the figure is time-dependant, the coloring of the temperature differences should not be compared between months. The images give an indication of the spatial distribution of the temperature difference between 2022 and the average for any one particular month. Absolute differences vary between months – for example, in July values range from 3°C to 11°C compared with September ranges from −1° to +2°C.

These time comparisons were repeated for each of the climate variables and environmental parameters in the study and statistics extracted at three spatial extents: (i) southeast UK, (ii) county of Kent, and (iii) a 10 km spatial buffer around each 2022 event recorded in HIC. While all events in 2022 were assessed, we focus here on results associated with a specific heatwave (HIC event unique reference #1038), wildfire (HIC event unique reference #1094) and flash flood (HIC event unique reference #1047) in the Kent area. The scope was to systematically identify any potential anomalies and tipping points preceding these hazard occurrences.

### Quantification and statistical analysis

This paper does not contain or use any statistical testing at the time of publishing.
