# Supplementary material for: Assessing sustained high-heat multi-hazard events through earth observation and impact chain analysis in southeast UK
Source: iScience. 2026 Mar 24;29(4):115423. doi: 10.1016/j.isci.2026.115423 (PMC13091469; doi:10.1016/j.isci.2026.115423)
Supplement: Document S1. Tables S1–S3 [file mmc1.pdf]

iScience, Volume *29*

Supplemental information

Assessing sustained high-heat multi-hazard events through earth observation and impact chain analysis in southeast UK

Erin Mills, Kay Smith, Annie Winson, Luke Bateson, and Roxana Ciurean

Table S1

**Climate: ERA5-Land ECMWF Climate Reanalysis (1950-present)**	
Source	Google Earth Engine<br><br>Collection: ee.ImageCollection("ECMWF/ERA5_LAND/MONTHLY_AGGR")
Spatial Resolution	0.1° x 0.1° (exported as 1km)
Temporal Resolution	Monthly (aggregate)
File Format	GRIB (exported as GeoTIFF)
Citation	Muñoz Sabater, J., (2019): ERA5-Land monthly averaged data from 1981 to present. Copernicus Climate Change Service (C3S) Climate Data Store (CDS). (<date of access>), doi:10.24381/cds.68d2bb30

**Parameters**	
Air Temperature	Temperature (K) at 2m above the surface (land, sea or in-land water)<br><br>Variable: 'temperature_2m' (also *_max/*_min)
Precipitation	Total accumulated liquid and frozen water (m) that falls to the surface, accumulated across the time step, generated from large-scale weather patterns and convection from the surface, equivalent to the depth of water if spread across the grid cell.<br><br>Variable: 'total_precipitation_sum' (also *_max/*_min)
Soil Temperature	Temperature (K) at the middle of the soil layer (layer 1: 0-7cm; layer 2: 7-28cm; layer 3: 28-100cm; layer 4: 100-289cm) with heat transfer at the interface between layers with none transferring out of the lowest layer.<br><br>Variables: 'soil_temperature_level_1', 'soil_temperature_level_2', 'soil_temperature_level_3' and 'soil_temperature_level_4' (also *_max/*_min)
Soil Moisture	Volume of water (m3/m3) in the soil layers (layer 1: 0-7cm; layer 2: 7-28cm; layer 3: 28-100cm; layer 4: 100-289cm) associated with soil texture, soil depth and the underlying groundwater level.<br><br>Variables: 'volumetric_soil_water_layer_1', 'volumetric_soil_water_layer_2', 'volumetric_soil_water_layer_3' and 'volumetric_soil_water_layer_4' (also *_max/*_min)
Evaporation	Evaporation (m of water equivalent) from vegetation transpiration accumulated across the time step, same meaning as root extraction as the amount of water extracted from the different soil layers.<br><br>Variable: 'evaporation_from_vegetation_transpiration_sum' (also *_max/*_min)
Runoff	Total amount of water (m) drained away (surface and sub-surface) as a measure of the availability of water in the soil, accumulated across the time step, equivalent to the depth of water if spread across the grid cell.<br><br>Variable: 'runoff_sum' (also *_max/*_min)

Land Surface Temperature: MODIS (2000-present)	
Source	Google Earth Engine<br><br>MODIS Terra Collection: ee.ImageCollection("MODIS/061/MOD11A2")<br><br>MODIS Aqua Collection: ee.ImageCollection("MODIS/061/MYD11A2")
Spatial Resolution	1km
Temporal Resolution	8-day average (exported as 8-day averages and monthly aggregates)
File Format	HDF-EOS (exported as GeoTIFF)
Citation	DOI: 10.5067/MODIS/MOD11A2.061<br><br>DOI: 10.5067/MODIS/MYD11A2.061

Parameters	
Day Temperature	Temperature (K) on surface (land, sea or in-land water) as simple averaging of all daily land surface temperature values measured during satellite day overpass (10:30 for MOD11A2 and 13:30 for MYD11A2)<br><br>Variable: 'LST_Day_1km' - requires scale factor of 0.02
Night Temperature	Temperature (K) on surface (land, sea or in-land water) as simple averaging of all daily land surface temperature values measured during satellite night overpass (22:30 for MOD11A2 and 01:30 for MYD11A2)<br><br>Variable: 'LST_Night_1km' - requires scale factor of 0.02

Leaf Area Index/FPAR: MODIS (2000-present)	
Source	Google Earth Engine<br><br>MODIS Terra Collection: ee.ImageCollection("MODIS/061/MOD15A2H)<br><br>MODIS Aqua Collection: ee.ImageCollection("MODIS/061/MYD15A2H")
Spatial Resolution	500m
Temporal Resolution	8-day average (exported as 8-day averages and monthly aggregates)
File Format	HDF-EOS (exported as GeoTIFF)
Citation	DOI: 10.5067/MODIS/MOD15A2H.061<br><br>DOI: 10.5067/MODIS/MYD15A2H.061

Parameters

Leaf Area Index (LAI)	One-sided green leaf area per unit ground area (m2/m2) in broadleaf canopies OR one-half total needle surface area per unit ground area (m2/m2) in coniferous canopies, measured during satellite day overpass (10:30 for MOD11A2 and 13:30 for MYD11A2)<br><br>Variable: 'Lai_500m' - requires scale factor of 0.1
Fraction of Photosynthetically Active Radiation (FPAR)	Fraction (%) of incident photosynthetically active radiation (400-700nm) absorbed by green elements of the vegetation canopy, measured during satellite day overpass (10:30 for MOD11A2 and 13:30 for MYD11A2)<br><br>Variable: 'Fpar_500m' - requires scale factor of 0.01

**Net Photosynthesis: MODIS (2021-present)**	
Source	Google Earth Engine<br><br>MODIS Terra Collection: ee.ImageCollection("MODIS/061/MOD17A2H)<br><br>MODIS Aqua Collection: ee.ImageCollection("MODIS/061/MYD17A2H")
Spatial Resolution	500m
Temporal Resolution	8-day average (exported as 8-day averages and monthly aggregates)
File Format	HDF-EOS (exported as GeoTIFF)
Citation	DOI: 10.5067/MODIS/MOD17A2H.061<br><br>DOI: 10.5067/MODIS/MYD17A2H.061

**Parameters**	
Net Photosynthesis	The amount of carbon captured by plants in an ecosystem (kgC/m2) measured as Gross Primary Productivity minus the maintenance respiration.<br><br>Variable: 'PsnNet' - requires scale factor of 0.0001

**Population: Global Human Settlement Layer (1975-2023 projected)**	
Source	GHSL – Global Human Settlement Layer<br><br>https://human-settlement.emergency.copernicus.eu
Spatial Resolution	100m
Temporal Resolution	5-year epochs including 2018 @10m
File Format	GeoTIFF
Citation	Schiavina M., Melchiorri M., Pesaresi M., Politis P., Freire S., Maffenini L., Florio P., Ehrlich D., Goch K., Tommasi P., Kemper T., GHSL Data Package 2022, Publications Office of the European Union, Luxembourg, 2022, ISBN 978-92-76-53071-8, doi:10.2760/19817, JRC 129516

**Parameters**	
Built-up surface	Distribution of built-up surfaces estimates generated by spatial-temporal interpolation of multi-sensor/platform satellite images through radiometric, textural and morphological features within an unsupervised rule-based reasoning and inductive locally-adaptive method leveraging per-pixel spectral indices, multiscale textural fields assessments and object-oriented shape analysis.<br><br>Variable: 'GHS-BUILT-S'
Built-up height	Building height derived from ALOS Global Digital Surface Model (AW3D30) and NASA Shuttle Radar Topography Mission (SRTM) using linear regression.<br><br>Variable: 'GHS-BUILT-H'
Population	Distribution of human population (number of people per cell) derived from raw global census data harmonised by CIESIN for Gridded Population of the World (GPWv4.11) polygons, disaggregated from census to administrative units to grid cells, informed by the distribution, classification and density of built-up as mapped in the GHSL global layers per corresponding epoch.<br><br>Variable: 'GHS-POP'

Table S2

Hazard Type	Variable<br><br>(Climatic / Environmental)	Precondition		Baseline		Compound		Additional Comments
		Duration	Value	Duration	Value	Duration	Value	
Drought<br><br>(Kent-wide), 12/08/2022	Air Temperature (°C)	~30 days	>17.5°C	~35 days	>20°C	~3 days<br>~3 days	>23°C<br>>26°C	-Peak soil moisture prior to event was 0.36 on 6/06, again corresponding to precipitation event<br>-Lowest on 19/07 with 0.15, corresponding with peaks in both air and soil temperatures
	Soil Temperature (°C)	~30 days	>16.5°C	~25 days	>20°C	~10 days<br>~4 days	>23.5°C<br>>27.5°C	-Peak in air temperature on 19/07 with 35.2°C, and 30.7°C on 14/08 prior to event on 18/08<br>-Lowest occurred on 6/06 with 16.8°C, of which it remains above for subset period
	Soil Moisture (Volumetric Fraction)	~30 days	<0.45	~20 days	<0.3	~15 days	<0.2	-Largest peak and deviation from the average on 18/06 with 6.8mm, corresponding with rapid decline of air temperature and peak in soil moisture<br>-Longest period with precipitation consistently below <0.01mm leading up to the event was 11 days (3/08 to 14/08)<br>-Several smaller precipitation spikes (all <1.8mm), which all correspond with sudden declines in air temperatures
	Precipitation (mm)	~15 days	<10mm	~40 days	<3mm	~5 days<br>~5 days	<2mm<br><0.01mm	-Stable NDVI from 1/06 to 20/06, then begins to gradually decline, corresponding with gradual increase in both air and LST<br>-Increases between 10/07 and 20/07, associated with precipitation event and small increase in soil moisture

								-Stable leading up to event, ranging from 0.52-0.54 from 31/07 onwards
Heatwave (#1038), 11/08/2022	Air Temperature (°C)	~55 days	>15°C	~10 days	>19°C	5 days	>21°C	-Peak in LAI at start of observation period with 5.7 on 1/06, declining rapidly to 2.4 on 10/06, corresponding to gradual increase in LST & DMP<br>-From 10/06 to 18/08 when the event occurs, peak of 2.8 occurred on 26/06, just lagging from peaks in both precipitation and soil moisture<br>-Gradual increase from 1.0 on 1/08 to 2.0 on 13/08 just prior to event
	Land Surface Temperature (°C)	~30 days	>23°C	~20 days	>27°C	~10 days	>31°C	-Gradual increase from 97 on 1/06 to 149 on 20/06, corresponding with increasing air, soil and LST, in addition to decrease in soil moisture and LAI<br>-Decline from 30/06 to 31/07, and increase prior to event from 73 on 31/07 to 90 on 10/08
	Soil Temperature (°C)	~35 days	>16°C	~20 days	>21°C	~10 days	>24°C	-Peak at start of observation period of 0.91 on 1/06, sharply declining to 0.71 on 10/06<br>-Lowest measurement of 0.48 on 12/07 after several small peaks, which correspond with drops in air temperature and increases in precipitation
	Soil Moisture (Volumetric Fraction)	~20 days	<0.4	~15 days	<0.3	~30 days	<0.2	-Peak air temp of 32°C on 26/07 with sharp decline to 19.8°C on 29/07, then rising again prior to heatwave declaration<br>-Lowest temperature recorded during this temporal period was 17°C on 5/06 at the start of the time series, with a gradually increasing variable trend leading up to the event

								-Drop in temperatures corresponding with small precipitation peaks
Wildfire<br><br>(#1094), 18/08/2022	Air Temperature (°C)	~55 days	>16.5°C	~10 days	>19°C	~4 days<br><br>~5 days	>22°C<br><br>>26°C	-LST remains above 31°C throughout the observation period prior to the onset of the event on 6/08, with peak of 40.5°C recorded on 20/07<br><br>-Lowest recorded observation throughout the temporal period occurred after the event on 22/09 with 22.2°C, with significant daily variation leading up to this point, correlating with rapid dip in soil temperatures and spike in precipitation<br><br>-Drop in LST corresponding to small precipitation peaks, and sharp declines in both air and soil temp<br><br>-LST experiences drop in the immediate aftermath of heatwave declaration, corresponding to dips in both air and soil temps, and small peaks in both precipitation and resultant soil moisture
	Precipitation (mm)	~20 days	<7mm	~30 days	<2mm	~10 days<br><br>~10 days	<0.6mm<br><br><0.01mm	-Remains above 17°C throughout observation period prior to event on 6/08, but above 14°C for the entire temporal period<br><br>-sharp declines corresponding to dipping air and LST temps, and peaks in both precipitation and soil moisture
	NDVI	~15 days	>0.65	~40 days	>0.53	~15 days	<0.52	-Peaks in soil moisture correspond to peaks in precipitation and declines in both air and soil temps, with largest peak after the onset of event of 0.42 on 23/09<br><br>-Gradual decline in soil moisture at the beginning of the observational period associated with sustained increases in air and soil temps, declining from 0.36 on1/06 to 0.14 on 26/07
	LAI	~20 days	>1.5	~10 days	<2.5	~40 days<br><br>~5 days	<1.9<br><br><1.5	-Remains above 16.5°C prior to event on 17/07, and above 13.4°C for the remainder of the temporal period

								-Corresponding to increasing soil temps and dry matter productivity, declining soil moisture and below average precipitation<br><br>-maximum observation of 30.9°C on 27/07 and minimum of 13.4°C on 23/09, with corresponding patterns of soil temps
	Dry Matter Productivity (kg/ha/day)	~35 days	>47kg/ha/day	~35 days	>70.5kg/ha/day			-Remains below 2.8mm prior to event on 17/07, below from 1/06 through to 7/08<br><br>-Small visible peaks in precipitation corresponding with rapid dips in air temp and delayed spikes in soil moisture<br><br>-Below 0.02mm from 18/06 to 3/07 prior to event, linking to rapid increase in dry matter productivity yet relatively stable LAI
	Fraction Photosynthetically Active Radiation	~10 days	<0.87	~40 days	<0.73	~15 days<br><br>~5 days	<0.63<br><br><0.59	-Stability of trend prior to the event with maximum of 0.76 on 30/06, then gradually dipping to 0.6 at the time of the event<br><br>-The declining trend continues after the onset of the event, before levelling off and increasing from 10/08 onwards, with observation period peak of 0.85 on 31/08<br><br>-Peaks and trends slightly lag as a response of precipitation and soil moisture peaks, with declines seen alongside dips in air and soil temperatures
Flash Flood (#1047), 18/08/2022	Air Temperature (°C)	~78 days	>16.9°C	~47 days<br>~22 days	>19°C >21°C	~9 days	>24°C	-Temperature peak of 36.5°C on 19/07, and again of 31.2°C on 13/08 just prior to event on 18/08<br><br>-Rapid decline in the days preceding the flash flood associated with the reported thunderstorms that brought the rain
	Soil Temperature (°C)	~78 days	>17°C	~49 days	>19.5°C	~23 days	>21.5°C	-Peak of 35.2°C on 19/07, corresponding with spikes in air temp, and occurs just after LST peak of 33.9°C on 12/07 and another

								peak of 33.1°C on 14/08 just prior to event, declines sharply to 23.7 on 17/08
	Soil Moisture (volumetric Fraction)	~78 days	<0.35	~54 days	<0.29	~21 days	<0.17	-Small peaks corresponding with peaks in precipitation & slight drops in air temp<br>-Large, sharp increase corresponding with precipitation event on 18/08
	Precipitation (mm)	~78 days	<5mm	~50 days<br>~29 days	<1.1mm<br><0.5mm	~16 days	<0.01mm	-Precipitation recorded on 16/08 just prior to reported event with 3.5mm recorded, which could correspond to a lag in reporting
Heatwave<br>(#1118), 6/08/2018	Air Temperature (°C)	~66 days	>17°C	~49 days<br>~25 days	>19°C >22°C	~4 days	>26°C	-Relatively stable trend prior to the event, above 6.4 until rapidly dips to 4.7 on 28/07 in the aftermath of the event, corresponding to declines in FPAR and dry matter<br>-Minimum measurement throughout the observation period was 2 on 18/09, while maximum was 7 across several observation dates in the time series<br>-Large scale variation is focused in the latter stages of the temporal period, after the event occurred
	Land Surface Temperature (°C)	~65 days	>23°C	~32 days	>27°C	~8 days	>31°C	-Stable from 1/06 through to 10/06 with 138, then dips to 125 on 20/06, before rapidly rising again to the temporal period peak of 191 on 30/06 just prior to the event<br>-From 30/06 onwards the negative trend and small peaks are consistent with peaks in precipitation and soil moisture, as well as stability in FPAR and LAI<br>-The trend does not seem to accelerate after the occurrence of the event observed in this temporal window

	Soil Temperature (°C)	~48 days	>20°C	~39 days	>24°C	~4 days	>26°C	-Maximum of 1 throughout the time series, only dipping to minimum of 0.78 towards the tail end of the observational period<br><br>-Trend can be seen to dip after the occurrence of the vent on 17/07, from 1 on 16/07 to 0.87 on 28/07<br><br>-Corresponds well to low precipitation and resultant soil moisture, high LST and air/soil temps and decline in FPAR, NDVI & LAI<br><br>-Quick recovery seen from 0.87 on 28/07 to 1 on1/08 followed by a short period of relative stability
	Soil Moisture (Volumetric Fraction)	~65 days	<0.38	~55 days	<0.29	~5 days	<0.26	-Peak soil moisture prior to event was 0.36 on 6/06, again corresponding to precipitation event<br><br>-Lowest on 19/07 with 0.15, corresponding with peaks in both air and soil temperatures
Wildfire (#1106), 17/07/2018	Air Temperature (°C)	~46 days	>16.5°C	~24 days	>21°C	~5 days<br><br>~3 days	>23°C<br><br>>25°C	-Peak in air temperature on 19/07 with 35.2°C, and 30.7°C on 14/08 prior to event on 18/08<br><br>-Lowest occurred on 6/06 with 16.8°C, of which it remains above for subset period
	Precipitation (mm)	~46 days	<2.8mm	~11 days	<0.8mm	~3 days	<0.3mm	-Largest peak and deviation from the average on 18/06 with 6.8mm, corresponding with rapid decline of air temperature and peak in soil moisture<br><br>-Longest period with precipitation consistently below <0.01mm leading up to the event was 11 days (3/08 to 14/08)<br><br>-Several smaller precipitation spikes (all <1.8mm), which all correspond with sudden declines in air temperatures

	NDVI	~46 days	>0.68	~17 days	0.75			-Stable NDVI from 1/06 to 20/06, then begins to gradually decline, corresponding with gradual increase in both air and LST<br>-Increases between 10/07 and 20/07, associated with precipitation event and small increase in soil moisture<br>-Stable leading up to event, ranging from 0.52-0.54 from 31/07 onwards
	LAI	~46 days	>6.4	~8 days	<7.0			-Peak in LAI at start of observation period with 5.7 on 1/06, declining rapidly to 2.4 on 10/06, corresponding to gradual increase in LST & DMP<br>-From 10/06 to 18/08 when the event occurs, peak of 2.8 occurred on 26/06, just lagging from peaks in both precipitation and soil moisture<br>-Gradual increase from 1.0 on 1/08 to 2.0 on 13/08 just prior to event
	Dry Matter Productivity (kg/ha/day)	~27 days	>125kg/ha/day	~17 days	>191kg/ha/day	~7 days	<169kg/ha/day	-Gradual increase from 97 on 1/06 to 149 on 20/06, corresponding with increasing air, soil and LST, in addition to decrease in soil moisture and LAI<br>-Decline from 30/06 to 31/07, and increase prior to event from 73 on 31/07 to 90 on 10/08
	Fraction Photosynthetically Active Radiation	~13 days	>0.93	~9 days	=1			-Peak at start of observation period of 0.91 on 1/06, sharply declining to 0.71 on 10/06<br>-Lowest measurement of 0.48 on 12/07 after several small peaks, which correspond with drops in air temperature and increases in precipitation
	Air Temperature (°C)	~35 days	>17°C	~15 days	>20°C	~12 days	>22°C	-Gradual increase in temp leading up to event, with highly variable declining trend post-event onset

Flash Flood (#1119), 6/07/2018								-Peak of 28.3°C on 1/07 prior to event, with post-event peak of 30.3°C on 27/07<br><br>-Sharp decline from 28.8°C on 8/07 just after event to 21.9 °C on 11/07
	Soil Temperature (°C)	~35 days	>18.5	~15 days	>21.5°C	~9 days	> 24.5°C	-Peak of 27.4°C on 2/07 prior to event onset, with highly variable trend post-event<br><br>-Sharp decline from 28.7°C on 8/07 to 23.5°C on 11/07 in the event aftermath, corresponding to drop in air temp and onset of precipitation
	Soil Moisture (Volumetric Fraction)	~35 days	<0.4	~25 days	<0.3	~10 days	<0.2	-Declining trend associated with increasing air and soil temps, with very little precipitation peaks prior to onset<br><br>-Event peak of 0.91 on 7/07 during onset of precipitation event, with larger peaks later in the time series associated with increased ability of penetration and in turn saturation
	Precipitation (mm)	~35 days	<1.3mm	~19 days	<0.5mm	~15 days	<0.006mm	-Peak precipitation corresponding to onset of event with 2.98mm recorded on 5/07, corresponding to peak in soil moisture and dips in air and soil temps<br><br>-Highly variable post-event, corresponding to generally declining temperature trends and gradual increases in soil moisture

Table S3

ref_no	event_type	previous_event	date	month	year	duration	stakeholders	location	impact	source
1005	Heatwave	High Heat	15	June	2022	weeks	Met Office, Local Council, NHS	Kent, England	met office issued a level 3 heat-health alert for Kent following on from the level 2 warning given the previous day, and highlights the action within the 'heatwave action' procedure stage which requires health and social services to target specific actions at high-risk groups, the warnings highlight that most heat-related deaths occur in the first 2 days of a heatwave and emergency services can easily be under pressure during these times	Source
1006	Heatwave	High Heat	17	June	2022	weeks	Met Office, UK Health Security Agency	Sussex, England	advice has been given to those in Sussex regarding how to keep safe during the declared heatwave with expected temperatures of 34C in southern England, overnight minimum temperatures of 18C, this comes after met office and UKHSA issued a level 3 heat-health alert for London/East/South East England as these temperatures are 'unusual' in June	Source
1008	Heatwave	High Heat	8	July	2022	weeks	Met Office, NHS	Sussex, England	NHS issued a health alert for Sussex ahead of the 30C heatwave forecast, and encouraged people to look out for those vulnerable around them who may be at higher risk of adverse implications caused by heat	Source
1009	Heatwave	High Heat	9	July	2022	weeks	Met Office, Local Council/Gov	Kent, England	Kent's public health chief issued a warning to vulnerable residents in conjunction with the level 3 weather alert given by the met office, Cunty Council warning to those who may be vulnerable to high temperatures	Source
1011	Heatwave	High Heat	11	July	2022	weeks	EA, Met Office, UK Health Security Agency, NHS	Kent, England	EA advised that people should use water wisely, Met Office predicting temperatures of 31C in Gravesend/30C in Rochester and Maidstone/29C in Sittingbourne/29C in Canterbury/28C in Tunbridge Wells and 27C in Ashford, Met Office also increased level 2 alert to level 3 for the south east	Source
1012	Heatwave	High Heat	12	July	2022	weeks	UK Health Security Agency, Emergency Services, Local Gov	SE England	an amber warning released by the met office in addition to a level 3 heat health alert from the UK health security agency, high temperatures recorded over the south east and warnings for those travelling to expect disruptions, and emergency services admitting they are stretched with resources during this time	Source

1115	Landslide	High Heat	13	July	2022	days		Seaford Head, Sussex, England	Identified in BGS landslide database (21025), occurred on coast between Seaford Head and Hope Gap	Source
1017	Heatwave	High Heat	15	July	2022	weeks	Fire & rescue, NHS, local Gov, Public Health, UK Health Security Agency	Kent & Sussex, England	increased number of calls to emergency services, with UK Health Security agency responded by upgrading the heat alert to level 4, temperatures expected to reach 40C in some areas and break existing records set in 2003, the nights near tropical night conditions as temperatures not falling below mid-20s, warnings for transport networks as disruption is imminent due to potential power cuts,	Source
1018	Heatwave	High Heat	16	July	2022	weeks	Water Companies	Kent, England	Southern Water had power issues due to a reservoir failure - which serves the area of Broadstairs & St Peters, around 2000 homes were affected, the situation was exacerbated by the high temperatures and lack of recent precipitation in addition to the increased water usage	Source
1027	Heatwave	High Heat	18	July	2022	weeks	Met Office, fire & rescue service, Network Rail	South East England	met office issued red warning due to the extreme heat forecast which covers North Kent and parts of Surrey, which meant widespread impacts on people and infrastructure was expected with substantial changes in working practices and daily routines, while the rest of the South east was covered by an amber warning for extreme heat, several Kent residents were left with low water pressure due to increased demand associated with the warm weather, Network Rail advised that people should only travel 'is absolutely necessary' in the following days, drivers were warned to watch engine temperatures - particularly in older vehicles	Sources
